# Rational design of click-assembled chiral dendrimers: anticancer activity and molecular dynamics study

**DOI:** 10.1039/d6ra00668j

**Published:** 2026-03-02

**Authors:** Tamer El Malah, Ahmed A. El-Rashedy

**Affiliations:** a Photochemistry Department, Chemical Industries Research Institute, National Research Centre 33 El Buhouth Street, P.O. Box 12622 Cairo Egypt tmara_nrc3000@yahoo.com; b Chemistry of Natural and Microbial Products Department, National Research Centre Dokki 12622 Cairo Egypt; c Department of Organic and Medicinal Chemistry, Faculty of Pharmacy, University of Sadat City Monofia 32897 Egypt

## Abstract

This study details the rational design, synthesis, and biological evaluation of a range of chiral dendritic compounds created using modular copper(i)-catalyzed azide–alkyne cycloaddition (CuAAC) click chemistry. We developed first-generation dendrimers (6–9) with systematically different degrees of chirality, comprising fully chiral, achiral, and mixed-chirality systems, as well as a multivalent second-generation dendrimer (12). The biological screening against human cancer cell lines (HCT-116, HepG-2, and MCF-7) revealed that the fully chiral first-generation dendrimer (9) was the most effective. Significantly, dendrimer (9) presented improved selectivity, as evidenced by a favorable therapeutic window with considerably reduced toxicity to normal WI-38 fibroblasts (IC_50_ = 46.79 ± 2.8 µM) relative to the reference drugs doxorubicin and sorafenib. On the other hand, the second-generation dendrimer (12) revealed slight cytotoxic effects, which can be attributed to limited cellular absorption related to its larger molecular size. Molecular dynamics (MD) simulations conducted on the ERα receptor have verified that dendrimer (9) establishes a stable complex with a total binding free energy (Δ*G*_bind_) of −65.07 ± 0.20 kcal mol^−1^, which is mainly influenced by robust van der Waals interactions and hydrophobic packing. Moreover, frontier molecular orbital (FMO) analysis has characterized dendrimer (9) as a kinetically stable entity with a HOMO–LUMO energy gap of 2.74 eV. These observations emphasize the important role of chirality and dendritic generation in anticancer potency, positioning these click-assembled chiral frameworks as promising lead structures for further development in targeted cancer therapy.

## Abstract 2

This study describes the design, synthesis, and evaluation of chiral dendrimers using click chemistry. The fully chiral dendrimer showed the highest anticancer activity, good selectivity, stable ERα binding, and promising potential for targeted cancer therapy.

## Graphical abstract

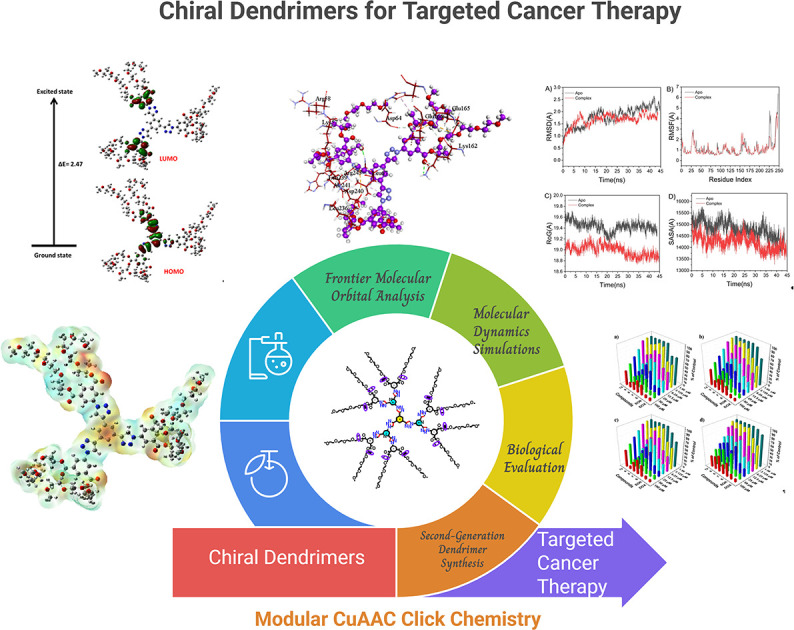

## 1. Introduction

Cancer remains a major global health challenge, accounting for nearly 10 million deaths each year and creating substantial socioeconomic burdens on healthcare systems worldwide.^1^ Despite significant advancements in the understanding of cancer biology and the development of therapeutic strategies,^2^ the identification of anticancer agents that effectively combine high efficacy with selective toxicity towards malignant cells continues to be a primary challenge in medicinal chemistry and pharmaceutical research.^3^ The biological complexity and heterogeneity of cancer necessitate innovative molecular designs that can achieve selective recognition and targeted interactions with cancer cells, while also minimizing harm to normal tissues.^4^ In this context, nanostructured systems have emerged as promising alternatives to standard small-molecule chemotherapeutics. Among these, dendrimers have gained significant attention due to their highly branched three-dimensional architecture,^5^ uniformity, and the presence of multiple peripheral functional groups that can be precisely adjusted. The architecture of dendrimers, which varies with generation, allows for fine control over molecular size, surface charge density, and the spatial arrangement of functional moieties, which in turn affects cellular uptake, biodistribution, and target specificity.^6^ These features make dendrimers appealing platforms not only for drug delivery but also as standalone multifunctional therapeutic agents.^7^ The development of dendritic structures has been greatly enhanced through the use of click chemistry, especially the copper(i)-catalyzed azide–alkyne cycloaddition (CuAAC).^8^ This reaction provides exceptional chemoselectivity, remarkable efficiency, mild reaction conditions, and extensive functional group tolerance, facilitating the modular and reproducible creation of intricate dendrimers with high structural accuracy.^9^ Notably, the resulting 1,2,3-triazole linkages serve not only as strong structural connectors but also exhibit advantageous pharmacological properties, such as chemical stability, resistance to metabolic breakdown, and the capacity to engage in hydrogen bonding and other noncovalent interactions with biological targets.^10,11^ Another essential aspect of anticancer drug design is chirality, which is crucial in molecular recognition processes within biological systems.^12^ Chiral discrimination significantly affects drug-receptor binding, cellular internalization pathways, and pharmacokinetic behavior.^13^ The intentional integration and systematic adjustment of chiral units within dendritic frameworks offer a robust approach to explore structure-activity relationships and to refine biological responses.^14^ Furthermore, in multivalent dendritic systems, the spatially organized display of various chiral motifs may lead to cooperative binding effects, potentially improving selectivity and anticancer efficacy.^15^ The therapeutic potential of anticancer agents is primarily influenced by their ability to selectively suppress cancer cell growth while minimizing cytotoxic effects on normal cells.^16^ Although standard chemotherapeutic agents, such as doxorubicin and other anthracyclines, show significant anticancer activity, their clinical application is frequently limited by serious dose-dependent toxicity, poor tumor selectivity, and the rise of multidrug resistance.^17^ These challenges further highlight the importance of developing innovative molecular frameworks that can enhance the therapeutic window *via* increased selectivity. Consequently, chiral, click-assembled dendritic architectures have attracted growing interest as multifunctional anticancer systems that combine nanoscale organization, multivalency, triazole-based connectivity, and stereochemical control.^18,19^ These platforms present a persuasive strategy for enhancing cancer cell selectivity while reducing negative impacts on normal tissues.^20^ Several dendrimer classes have demonstrated inherent cytotoxicity, independent of drug-loading, through mechanisms such as membrane disruption *via* cationic surface groups, induction of oxidative stress, or interaction with intracellular targets.^21^ For instance, poly(amidoamine) (PAMAM) and poly(propylene imine) (PPI) dendrimers can exhibit generation- and surface charge-dependent toxicity, often correlating with non-specific cell membrane interaction.^22^ The novelty of the present system lies in its deliberate integration of precisely controlled chirality and click-assembled triazole linkages within a dendritic architecture to modulate biological activity. Unlike many prior cytotoxic dendrimers whose activity stems largely from polycationic surfaces or conjugated toxins, our design investigates how stereochemical organization and multivalent presentation of bioinert, metabolically stable triazole linkers can influence cancer cell selectivity.^23^ This approach shifts the focus from generic membrane perturbation to potential stereoselective target recognition, offering a distinct strategy for developing dendrimers as standalone therapeutic agents with tunable selectivity. In this study, we detail the rational design, synthesis, and biological evaluation of chiral dendritic compounds created through modular CuAAC click chemistry.^24,25^ We synthesized first-generation dendrimers with systematically altered degrees of chirality by employing chiral and achiral dendrons alongside a trifunctional core scaffold. This method allowed for the development of fully chiral, achiral, and mixed-chirality systems, which are essential for thorough structure-activity relationship analysis. Additionally, a second-generation dendrimer with greater molecular complexity and multivalency was assembled through a convergent strategy to examine generation-dependent biological effects. The anticancer efficacy of the synthesized dendrimers was assessed against a variety of human cancer cell lines, which included colorectal carcinoma (HCT-116), hepatocellular carcinoma (HepG-2), and breast adenocarcinoma (MCF-7). Selectivity was determined using normal human lung fibroblasts (WI-38), with a comparative analysis conducted against the reference drugs doxorubicin and sorafenib. This thorough evaluation seeks to clarify the influence of dendritic generation and chirality on anticancer effectiveness and selectivity, thus aiding in the advancement of optimized dendritic structures as potential therapeutic agents for cancer.

## 2. Results and discussion

### 2.1. Chemistry

**Scheme 1:**
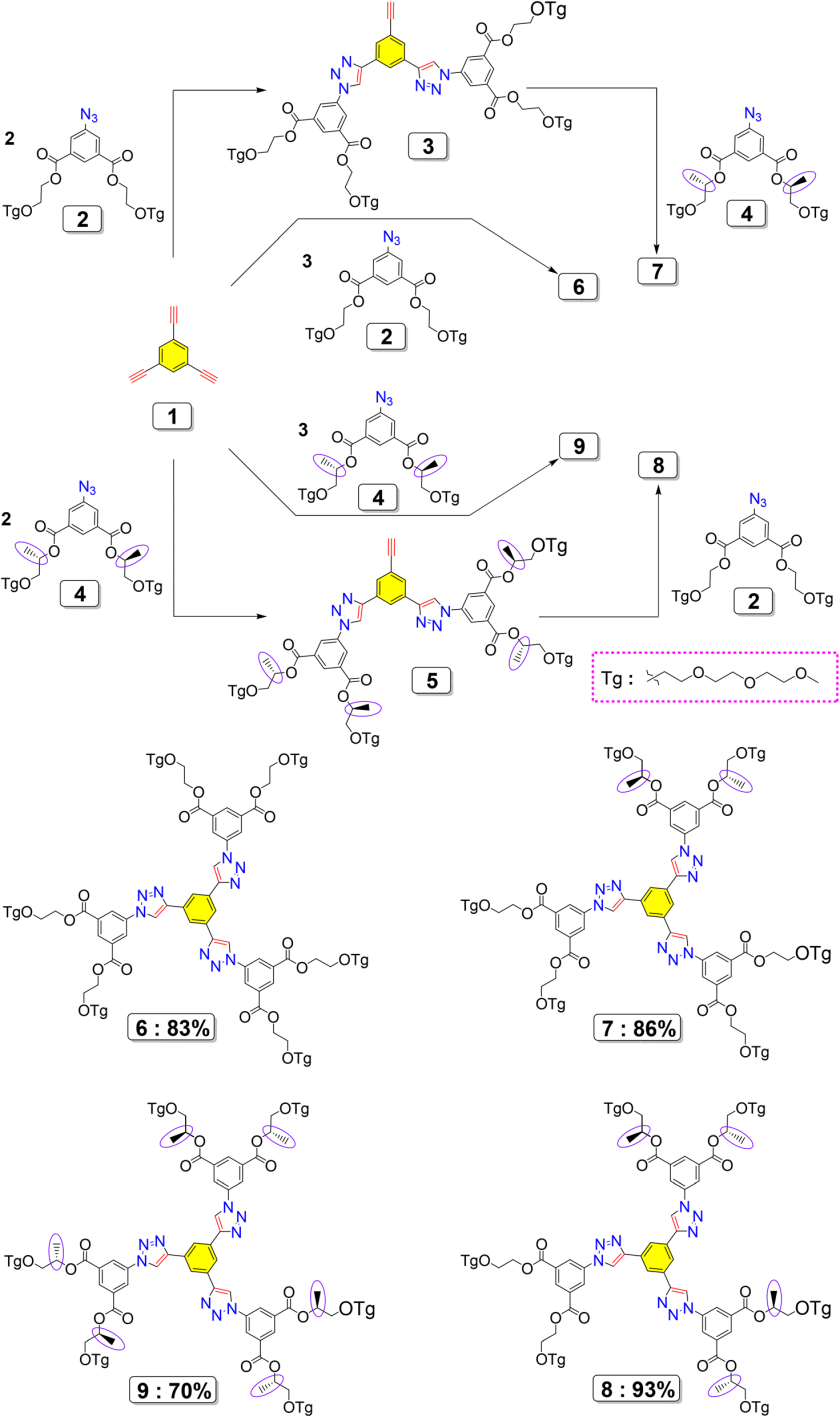
Synthesis of first-generation dendrimers with different degrees of chirality *via* click chemistry.

The first generation of chiral dendrimers, which exhibit four different degrees of chirality, was successfully synthesized and structurally characterized as shown in Scheme 1. The fully chiral dendrimer 9 (ref. 26) was achieved through a threefold Cu(i)-catalyzed azide–alkyne cycloaddition (CuAAC) reaction with chiral dendron 4. In contrast, the non-chiral analogue 6 was synthesized *via* a similar threefold click reaction using the achiral dendron 2. Mixed-chirality dendrimers 7 and 8 were synthesized through a stepwise approach that involved an initial twofold CuAAC reaction with dendrons 2 or 4, respectively, followed by a subsequent click reaction with the complementary dendron. This modular method provided precise control over the degree and distribution of chirality within the dendritic framework. All CuAAC reactions were performed according to a well-established protocol,^27^ which involved the *in situ* generation of Cu(i) from CuSO_4_ and sodium ascorbate. The reactions took place in the presence of tris[(1-benzyl-1*H*-1,2,3-triazol-4-yl)methyl]amine (TBTA) as a stabilizing ligand, utilizing a biphasic solvent system made up of aqueous tert-butanol and methylene chloride. This approach guaranteed high efficiency, chemoselectivity, and the structural integrity of the resulting dendrimers.

**Scheme 2:**
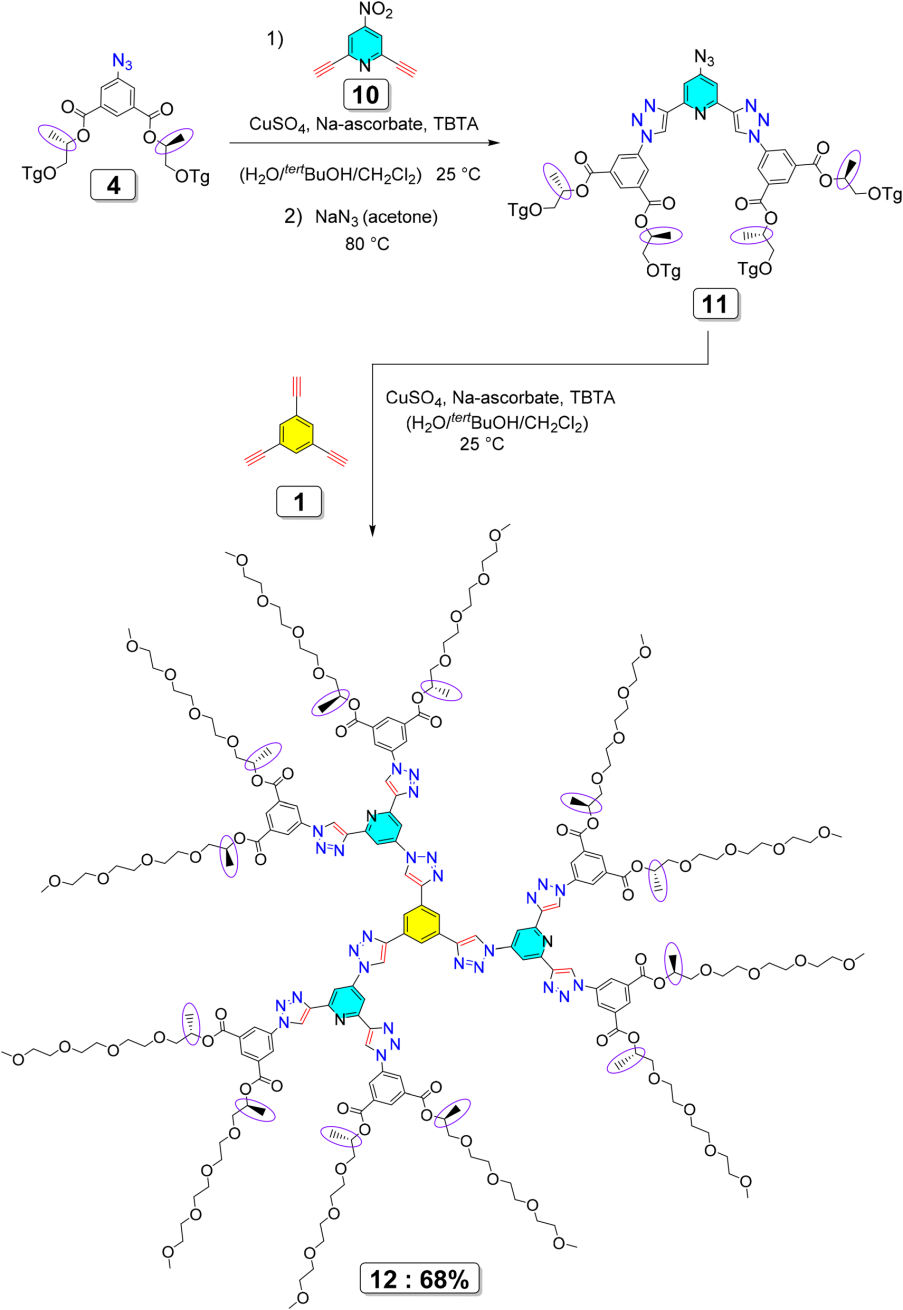
The convergent click-chemistry approach for synthesizing second-generation dendrimers.

Expanding on the synthetic logic introduced in the first generation, Scheme 2 (ref. 26) illustrates the advancement towards second-generation dendritic compounds, characterized by a significant rise in molecular size, structural intricacy, and multivalency. In the first step, azide derivative 4 participated in a CuAAC reaction with the multifunctional alkyne precursor 10 under standard click conditions^28^ (CuSO_4_/sodium ascorbate/TBTA), resulting in the formation of the triazole-linked intermediate 11. This intermediate serves as a pre-organized, multivalent scaffold that adds an extra hierarchical level to the dendritic architecture. The subsequent transformation of intermediate 11 through nucleophilic aromatic substitution with sodium azide produced reactive azide termini, which were then utilized in a convergent CuAAC reaction with the trifunctional core 1. This conclusive click assembly yielded the second-generation dendrimer 12 (ref. 26), characterized by a highly substituted, radially symmetric, dendritic-like design. The first-generation dendrimers were purposefully designed to control and analyze different levels of chirality and their impact on biological behavior, while the second-generation structure was chiefly aimed at increasing multivalency, spatial expansion, and surface functionalization. Crucially, the chiral information embedded in the first-generation motifs is maintained and multiplied throughout the higher-generation dendritic structure. This collaborative interaction between chirality and multivalency is anticipated to facilitate cooperative binding interactions with chiral cancer-associated biomolecular targets, thus improving biological recognition, binding selectivity, and ultimately leading to enhanced anticancer activity *via* increased cellular uptake and cytotoxic effectiveness.

### 2.2. Biology

#### 2.2.1. Anticancer activity

**Fig. 1:**
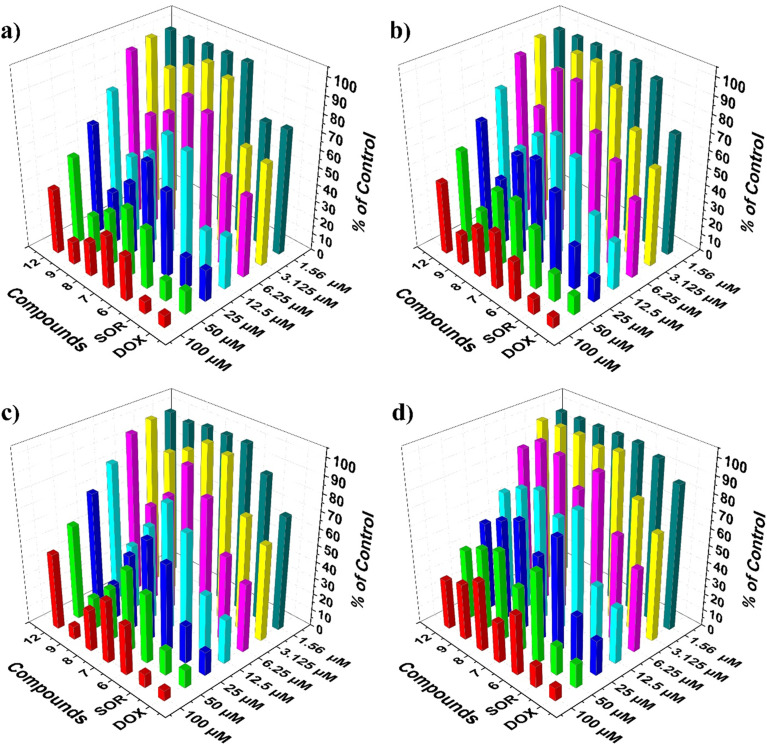
Cell viability percentage of the first generation dendrimer 6–9 and the second generation dendrimer 12 on the various cancer cell line types (a) HCT-116, (b) HePG-2, (c) MCF-7 and (d) WI38, using the MTT assay.

The anticancer effectiveness of the synthesized dendrimers belonging to the first generation dendrimer 6–9 and the second generation dendrimer 12 was assessed *in vitro* against three human cancer cell lines: HCT-116 (colon carcinoma), HepG-2 (hepatocellular carcinoma), and MCF-7 (breast adenocarcinoma). Additionally, the normal human lung fibroblast cell line WI-38 was included in the evaluation, utilizing the standard MTT colorimetric assay. For comparative analysis, doxorubicin (DOX) and sorafenib (SOR) were used as reference drugs. Cytotoxic activity was represented as IC_50_ values (µM), indicating the concentration needed to suppress 50% of cell proliferation after a treatment duration of 48 hours. The results are presented in Table 1 and Fig. 1. The screening findings demonstrated significant differences in cytotoxic activity influenced by the structural properties of the dendrimers and the tested cell line. Dendrimer 9 has emerged as the most powerful derivative across all three cancer cell lines, demonstrating strong cytotoxic effects with IC_50_ values of 12.93 ± 0.9 µM (HCT-116) and 16.18 ± 1.2 µM (HepG-2), while also showing the greatest selectivity towards MCF-7 breast cancer cells with an IC_50_ value of 9.78 ± 0.7 µM. When tested against normal WI-38 cells, dendrimer 9 exhibited an IC_50_ of 46.79 ± 2.8 µM, indicating a preference for cytotoxicity against cancer cells rather than normal cells. Dendrimer 8 also revealed promising anticancer activity, particularly against the HCT-116 (IC_50_ = 19.43 ± 1.4 µM) and MCF-7 (IC_50_ = 21.35 ± 1.4 µM) cell lines, thereby categorizing it as a strong anticancer agent. However, its activity against HepG-2 cells was moderate (IC_50_ = 37.71 ± 2.3 µM). The selectivity profile of dendrimer 8 was less favorable in comparison to dendrimer 9, which exhibited an IC_50_ value of 62.53 ± 3.5 µM against WI-38 cells. Dendrimer 6 demonstrated moderate cytotoxic effects across all evaluated cell lines, with IC_50_ values of 25.12 ± 1.6 µM for HepG-2, 28.62 ± 1.8 µM for HCT-116, and 32.75 ± 2.0 µM for MCF-7. Interestingly, dendrimer 6 showed comparatively lower toxicity towards normal WI-38 cells (IC_50_ = 57.41 ± 3.3 µM), indicating a favorable therapeutic window. Dendrimer 7 exhibited moderate activity against HCT-116 (IC_50_ = 38.21 ± 2.5 µM), HepG-2 (IC_50_ = 43.36 ± 2.6 µM), and MCF-7 cells (IC_50_ = 50.17 ± 2.9 µM). Importantly, dendrimer 7 showed improved selectivity for cancer cells, with the lowest toxicity observed in normal WI-38 cells (IC_50_ = 29.67 ± 1.9 µM) among the dendrimers evaluated. The second generation dendrimer 12 exhibited minimal cytotoxic effects on all cancer cell lines, with IC_50_ values of 55.37 ± 3.2 µM for HCT-116, 65.01 ± 3.4 µM for HepG-2, and 74.92 ± 3.8 µM for MCF-7. This diminished activity could be linked to its substantial molecular size and potentially restricted cellular absorption, though direct measurements of uptake were not performed in this study and remain to be investigated. Furthermore, dendrimer 12 demonstrated moderate toxicity towards normal WI-38 cells (IC_50_ = 34.84 ± 2.3 µM), suggesting that this dendrimer is relatively non-selective and exhibits similar cytotoxicity against both cancerous and non-cancerous cells. In comparison to the reference medications, dendrimers 8 and 9 showed IC_50_ values that were roughly 2–4 times greater than that of doxorubicin (DOX: IC_50_ = 4.17–5.23 µM) and were similar to those of sorafenib (SOR: IC_50_ = 5.47–9.18 µM). While the synthesized dendrimers were less potent than DOX in direct cytotoxicity comparisons, they demonstrated significantly improved selectivity profiles relative to both reference medications, especially dendrimer 9, which revealed significantly reduced toxicity towards normal cells. The observed cytotoxicity of dendrimer 9 across all three cancer cell lines suggests a broad-spectrum anticancer effect. While the molecular dynamics and docking analyses presented herein focus on its interaction with the estrogen receptor alpha (ERα) a highly relevant target for MCF-7 (breast adenocarcinoma) cells the activity against HCT-116 (colon carcinoma) and HepG-2 (hepatocellular carcinoma) likely involves alternative or additional mechanisms. These may include non-receptor-mediated pathways, such as disruption of mitochondrial function, induction of apoptosis *via* caspase activation, or interference with other signaling networks common in epithelial cancers. Further mechanistic studies are warranted to elucidate the specific targets and pathways involved in each cell line.

**Table 1:** Anticancer activity expressed as IC_50_ (µM/ml) of the first generation dendrimer 6–9 and the second generation dendrimer 12 on the four cancer cell types (HCT116, HePG2, MCF-7and WI38) relative to doxorubicin (DOX) and sorafenib (SOR)^a^

Entry	IC_50_ (µM) ± SD			
	HCT-116	HePG-2	MCF-7	WI-38
6	28.62 ± 1.8	25.12 ± 1.6	32.75 ± 2.0	57.41 ± 3.3
7	38.21 ± 2.5	43.36 ± 2.6	50.17 ± 2.9	29.67 ± 1.9
8	19.43 ± 1.4	37.71 ± 2.3	21.35 ± 1.4	62.53 ± 3.5
9	12.93 ± 0.9	16.18 ± 1.2	9.78 ± 0.7	46.79 ± 2.8
12	55.37 ± 3.2	65.01 ± 3.4	74.92 ± 3.8	34.84 ± 2.3
DOX	5.23 ± 0.3	4.50 ± 0.2	4.17 ± 0.2	6.72 ± 0.5
SOR	5.47 ± 0.3	9.18 ± 0.6	7.26 ± 0.3	10.65 ± 0.8

a IC_50_ (µM): 1–10 (very strong). 11–20 (strong). 21–50 (moderate). 51–100 (weak) and above 100 (non-cytotoxic) DOX: doxorubicin. SOR: sorafenib.

##### 2.2.1.1 Therapeutic significance of dendrimer 9

The enhanced selectivity and reduced toxicity of dendrimer 9 hold considerable clinical promise. Unlike conventional chemotherapeutics such as doxorubicin and sorafenib which exhibit potent cytotoxicity but often cause severe off-target effects due to poor selectivity dendrimer 9 demonstrates a favorable therapeutic window. Its IC_50_ value against normal WI-38 fibroblasts (46.79 µM) is substantially higher than those against cancer cell lines (9.78–16.18 µM), indicating preferential targeting of malignant cells. This selectivity stems from its fully chiral architecture, which promotes specific molecular recognition and binding to cancer-associated targets (*e.g.*, ERα) while minimizing interactions with healthy tissues. Such a profile could reduce dose-limiting toxicities, improve patient compliance, and mitigate the risk of drug resistance common limitations of traditional chemotherapy. Furthermore, the modular click-chemistry approach used to synthesize dendrimer 9 allows precise structural tuning, offering a versatile platform for developing next-generation, tumor selective anticancer agents with improved safety and efficacy.

#### 2.2.2. Discussion of selectivity mechanisms

The improved selectivity of dendrimer 9 its reduced toxicity toward normal WI-38 fibroblasts relative to cancer cell lines suggests a degree of preferential activity toward malignant cells. While the precise mechanistic basis requires further investigation, several structure-informed hypotheses can be proposed. First, the fully chiral, hydrophobic nature of dendrimer 9 may promote differential interactions with the altered lipid composition and higher membrane fluidity characteristic of cancer cell membranes, potentially facilitating preferential uptake. Second, the observed computational binding to ERα suggests a potential target-driven selectivity in ERα-positive MCF-7 cells, whereas non-specific cytotoxicity would likely affect normal and cancerous cells more equally. Third, cancer cells frequently exhibit enhanced metabolic activity and altered endocytic pathways, which could increase internalization of nanoscale dendrimeric structures. The fact that the less active dendrimer 6 (achiral) and the larger dendrimer 12 show reduced or non-selective cytotoxicity supports the hypothesis that both chirality and size are key determinants not just of potency but of selective recognition.

### 2.3. Molecular dynamic (MD) simulations

To gain preliminary mechanistic insight into the most active compound, dendrimer 9, we focused computational analyses on the estrogen receptor alpha (ERα) for several reasons: (1) ERα is a well-established therapeutic target in hormone-responsive breast cancers, including the MCF-7 cell line used in our cytotoxicity assays; (2) the receptor's well-characterized ligand-binding domain provides a suitable model for studying molecular recognition of chiral, hydrophobic compounds; and (3) previous studies have demonstrated that certain dendrimer architectures can interact with nuclear receptors through surface complementarity rather than traditional small-molecule binding modes. While this represents only one potential interaction among many, it provides a testable hypothesis for the selective activity observed in MCF-7 cells.

#### 2.3.1. Molecular dynamic and system stability

**Fig. 2:**
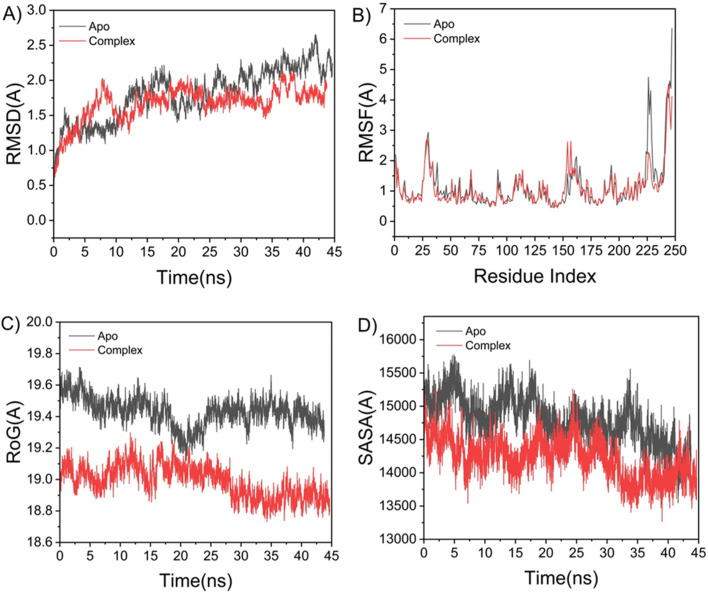
(A) RMSD of the protein backbone Cα atoms. (B) RMSF of each residue of the protein backbone Cα atoms of protein residues (C) ROG of Cα atoms of protein residues; (D) solvent accessible surface area (SASA) of the C α of the backbone atoms relative (black) to the starting minimized over 45 ns for the ATP binding site of ERα receptor with dendrimer 9 (red).

To gain insight into the potential mechanism of action of the most active compound, dendrimer 9, we conducted molecular docking and dynamics simulations against the estrogen receptor alpha (ERα). This target was selected due to its established role in breast cancer (MCF-7) and its relevance as a model system for studying ligand-receptor interactions in hormone-responsive malignancies. The following analyses are therefore interpreted primarily in the context of ERα-positive cancers, such as MCF-7, and are not intended to imply ERα as the exclusive or primary target in other tested cell lines (HCT-116 and HepG-2). Molecular dynamics (MD) simulations were performed to evaluate the binding behavior, interactions, and stability of the extracted dendrimers within the active site of the target protein.^29^ Validating system stability is essential for identifying genuine conformational motions and preventing the analysis of simulation artifacts. Detailed methodology for these simulations, including system preparation, minimization, equilibration, production runs, and subsequent analyses (MM/GBSA, DCCM, *etc.*), follows established protocols as described in Section 3.3. The overall stability of each system was assessed using the root-mean-square deviation (RMSD) of the protein backbone atoms over 45 ns of simulation. The ERα-9 complex showed lower RMSD (1.21 ± 0.17 Å) than apo-ERα (1.44 ± 0.23 Å), indicating enhanced conformational stability upon ligand binding (Fig. 2A). To examine local flexibility, residue-level fluctuations were quantified *via* the root-mean-square fluctuation (RMSF) over the 45 ns trajectory. The average RMSF values were 1.15 ± 0.23 Å for apo ERα and 1.21 ± 0.17 Å for the ERα-9 complex (Fig. 2B). The reduced fluctuations in the ligand-bound complex suggest that the inhibitor restricts local mobility upon binding, particularly in key active-site regions. The radius of gyration (*R*_g_) was calculated to evaluate overall protein compactness and structural rigidity throughout the 45 ns simulation. The average *R*_g_ values were 19.42 ± 0.09 Å for the apo system and 18.98 ± 0.10 Å for the ERα-9 complex (Fig. 2C). The lower *R*_g_ in the bound state reflects a more compact and rigid architecture upon ligand binding. Finally, the solvent-accessible surface area (SASA) was measured to monitor changes in the exposure of the hydrophobic core, which is critical for protein stability.^30^ The average SASA was 14 792.32 Å^2^ for apo ERα and 14 219.38 Å^2^ for the ligand–bound complex (Fig. 2D). The reduced SASA, together with the consistent trends in RMSD, RMSF, and *R*_g_, confirms that the ERα-9 complex remains structurally intact and tightly packed within the receptor's catalytic domain throughout the 45 ns simulation.

#### 2.3.2. Hydrogen bond formation between amino acid residues and the ligand

**Fig. 3:**
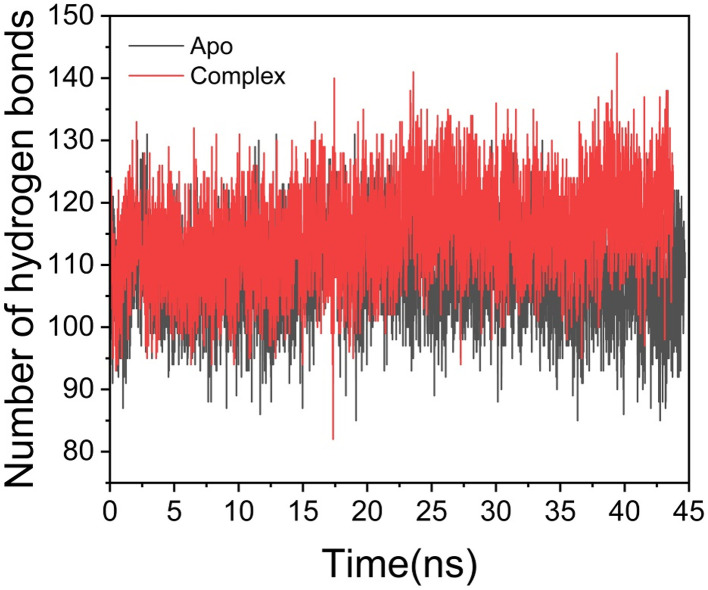
Number of hydrogen bond formation during simulation overtime between apo, and dendrimer 9-complex systems.

Hydrogen bonds play a critical role in biological systems, where they contribute to protein structural integrity, facilitate protein ligand interactions, and influence binding affinity and specificity.^31^ To assess the effect of ligand binding on conformational stability, we analyzed hydrogen bond formation throughout the simulation (Fig. 3). The apo system exhibited an average of 108.44 hydrogen bonds, while the ligand-bound complex maintained a higher average of 115.42. This reduction in hydrogen bond count within the apo form correlates with increased structural flexibility and decreased conformational stability, which may in turn affect the protein's capacity for ligand binding.

#### 2.3.3. Binding interaction mechanism based on binding free energy calculation

**Fig. 4:**
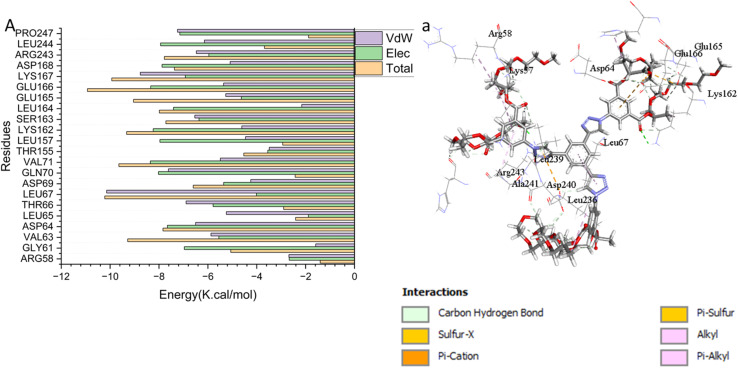
Per-residue decomposition plots showing the energy contributions to the binding and stabilization of dendrimer 9 to the ATP binding site of the Erα.

The binding free energies of the small molecules to the macromolecular target were calculated using the Molecular Mechanics Generalized Born Surface Area (MM/GBSA) method, a widely adopted approach for comparative and qualitative ranking of ligand binding affinities.^32^ It should be noted that while MM/GBSA provides valuable insights into the contributions of various energy terms (*e.g.*, van der Waals, electrostatic, solvation) and is often more reliable than docking scores, the absolute Δ*G*_bind_ values should be interpreted qualitatively rather than as precise experimental affinity predictions, especially for large, flexible systems such as dendrimers. Furthermore, the standard MM/GBSA approach typically does not include conformational entropy contributions, which can be significant for flexible ligands and large binding interfaces. Using the MM-GBSA module in AMBER18, binding free energies were computed from snapshots extracted from the production MD trajectories. As summarized in Table 1, the van der Waals (Δ*E*_vdW_), electrostatic (Δ*E*_ele_), and gas-phase interaction energies (Δ*E*_gas_) all contributed favorable (negative) values to the total binding affinity. The final binding free energy (Δ*G*_bind_) was derived from the sum of these gas-phase interactions and the solvation free energy (Δ*G*_solv_). The resulting Δ*G*_bind_ values indicate stable and favorable binding for the ligand protein complexes studied Fig. 4.

Table 2 presents the calculated binding energy components for dendrimer 9 when complexed with the ERα receptor, revealing a strong overall binding affinity of −65.07 ± 0.20 kcal mol^−1^. This favorable total binding free energy (Δ*G*_bind_) is primarily driven by a highly favorable van der Waals interaction (Δ*E*_vdW_ = −88.35 ± 1.05 kcal mol^−1^), indicating significant shape complementarity and hydrophobic packing within the receptor's binding pocket. In contrast, the electrostatic contribution (Δ*E*_elec_ = −3.80 ± 0.43 kcal mol^−1^) is modest, suggesting polar interactions play a secondary stabilizing role. The binding process is opposed by the solvation free energy (Δ*G*_solv_ = +23.28 ± 0.32 kcal mol^−1^), which reflects the thermodynamic penalty for desolvating the ligand and the binding site upon complex formation. Ultimately, the highly negative gas-phase interaction energy (Δ*G*_gas_ = −80.86 ± 0.05 kcal mol^−1^) outweighs this desolvation penalty, resulting in the net strong and stable binding observed for dendrimer 9. These results should be interpreted as indicative of a favorable and stable binding mode, with the large negative Δ*G*_bind_ value supporting the hypothesis that hydrophobic packing is the primary driver of the interaction. The strong, predominantly hydrophobic binding of dendrimer 9 to ERα suggests a potential mechanism for its observed cytotoxicity in MCF-7 cells. By occupying the ligand-binding domain with high affinity, dendrimer 9 could function as a steric inhibitor, potentially disrupting receptor dimerization, co-activator recruitment, or nuclear translocation key steps in ERα-mediated transcriptional activation. This could lead to downregulation of proliferation genes and induction of apoptosis in hormone-dependent cancer cells. It should be noted that this represents a hypothesized mechanism based on computational evidence; direct experimental validation of ERα pathway modulation is required to confirm this mode of action.

**Table 2:** Shows the calculated energy binding for dendrimer 9 against the ERα receptor^a^

Energy components (kcal mol^−1^)					
Complex	Δ*E*_vdW_	Δ*E*_elec_	Δ*G*_gas_	Δ*G*_solv_	Δ*G*_bind_
9-Complex	−88.35 ± 1.05	−3.80 ± 0.43	−92.15 ± 0.05	23.28 ± 0.32	−65.07 ± 0.20

a Δ*E*_vdW_ = van der Waals energy; Δ*E*_ele_ = electrostatic energy; Δ*G*_solv_ = solvation free energy; Δ*G*_bind_ = calculated total binding free energy.

#### 2.3.4. Identification of the critical residues responsible for ligands binding

**Fig. 5:**
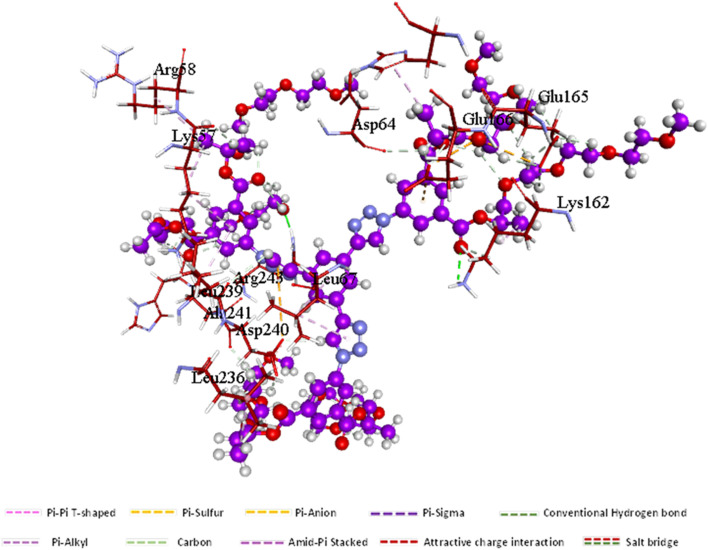
Key interactions between dendrimer 9 and the ERα receptor, highlighting hydrogen bonds and hydrophobic contacts.

To elucidate the specific residue-level contributions to binding, the total binding energy for dendrimer 9 was decomposed per residue within the ATP-binding site of the ERα receptor. This analysis identifies key amino acids responsible for favorable interactions with the ligand. The most significant favorable contributions (Δ*G* < −4.0 kcal mol^−1^) are listed in SI Table S1. These residues, which form critical hydrophobic, electrostatic, and hydrogen-bonding interactions (see also Fig. 5), collectively stabilize the ligand within the binding pocket and are central to the inhibitory mechanism. It worth to noted that the Residue numbers correspond to the AMBER-prepared simulation system and differ from the canonical PDB (3ERT) numbering.

#### 2.3.5. Ligand–residue interaction network profiles

##### 2.3.5.1 Docked 9 dendrimer – ERα complex

Dendrimer 9, which occupies the catalytic active site of ERα (Fig. 5), forms several key interactions with the protein. A hydrogen bond is observed with Arg243. Additionally, the dendrimer engages in hydrophobic π-alkyl interactions with Leu263, Leu67, and His169. Notably, the pharmacophoric hotspot residue Lys162 participates in multiple interactions, including a hydrogen bond, an alkyl interaction, and a carbon–hydrogen bond with the ligand.

#### 2.3.6. Dynamics cross-correlation matrices (DCCM) analysis

**Fig. 6:**
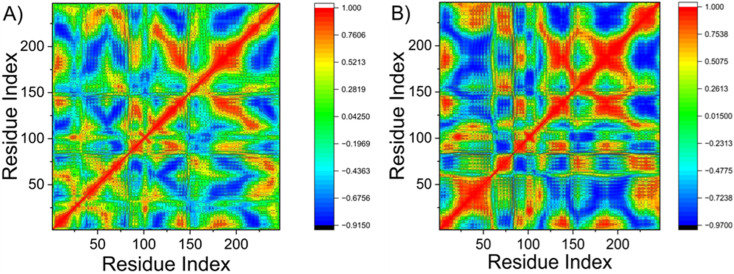
The dynamic cross-correlation matrix (DCCM) analysis, showing correlated (A) and anti-correlated motions upon ligand binding (B).

To investigate the impact of ligand binding on protein dynamics, dynamic cross-correlation matrices (DCCM) were calculated for the Cα atoms across the simulation trajectories. This analysis quantifies the correlated and anti-correlated motions between residue pairs, providing insight into conformational changes and allosteric communication within ERα. The DCCM plot (Fig. 5) reveals the overall motion patterns, where warm colors (yellow to red) indicate positively correlated motions (residues moving in the same direction), and cool colors (blue to black) represent anti-correlated motions (residues moving in opposite directions). Analysis of the ERα-9 complex indicates a predominance of positively correlated motions across the structure. As shown in Fig. 5, strong positive correlations are localized within two key regions: residues 0–50 and 180–248. Notably, the latter region encompasses the hydrophobic core of the catalytic active site, suggesting that ligand binding enhances cooperative dynamics among these functionally important residues. This coordinated motion likely stabilizes the active site architecture upon inhibitor binding (Fig. 6).

#### 2.3.7. Free energy landscape (FEL) analysis

**Fig. 7:**
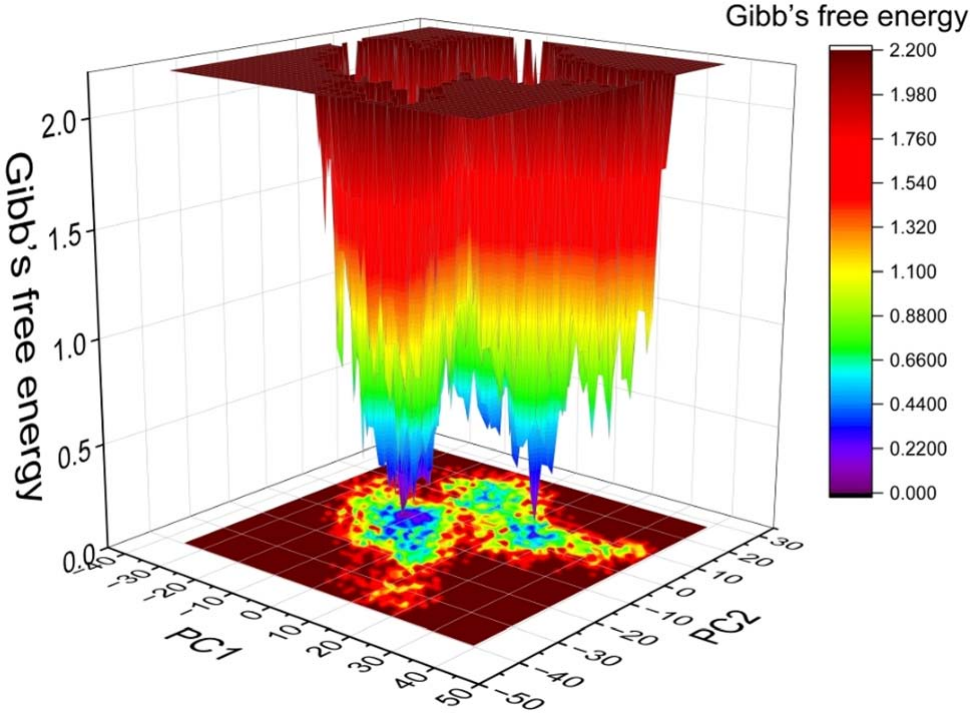
Free energy landscape (FEL) of ERα-dendrimer 9 complex.

As the stability of the conformation of proteins is associated with lower Gibb's free energy values, the PCA-based free energy landscape (FEL) was performed to evaluate the stability of protein conformations for the simulated complexes that has been provided in Fig. 7. From the FEL analysis, it was observed that the simulated complex was able to form stable conformation with low Gibb's free energy (blue and violet, Fig. 7).

#### 2.3.8. Probability density function (PDF) analysis

**Fig. 8:**
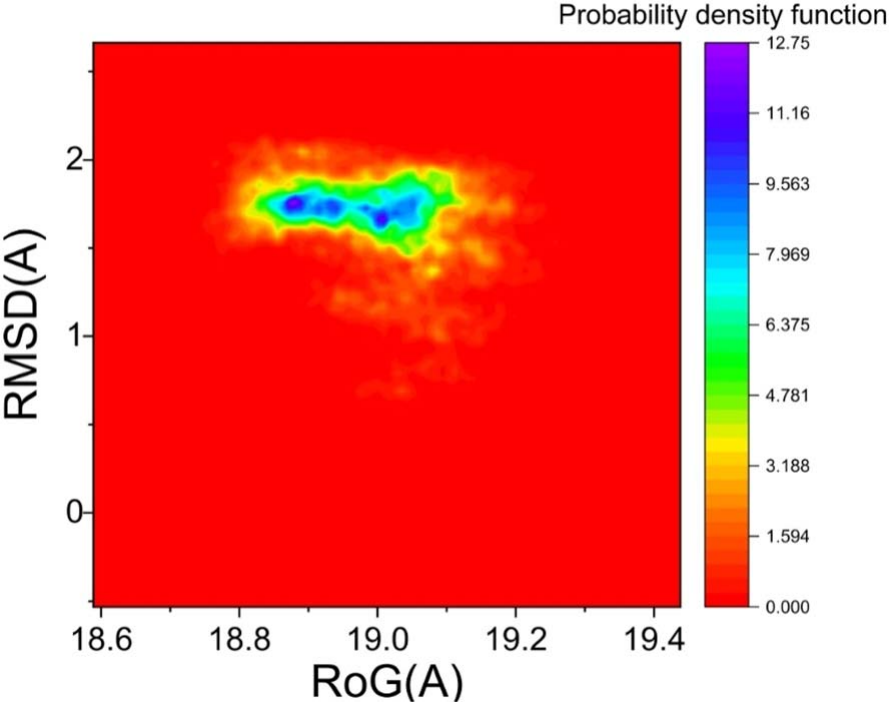
The free energy landscape (FEL) analysis, providing insights into the conformational stability of the ERα-dendrimer 9 complex.

The probability density function (PDF) analysis indicates the likelihood of occurrences of protein trajectories based on KDE(36) the radius of gyration (*R*_g_) and RMSD-based PDF plots for the ERα-dendrimer 9 complex is provided in Fig. 8. Interestingly, the PDF analysis for the ERα-dendrimer 9 complexes suggested that the largest populated conformations can be found with the *R*_g_ value of 18.93 Å and RMSD value of 1.73 Å (Fig. 8).

#### 2.3.9. Frontier molecular orbitals (FMOs)

**Fig. 9:**
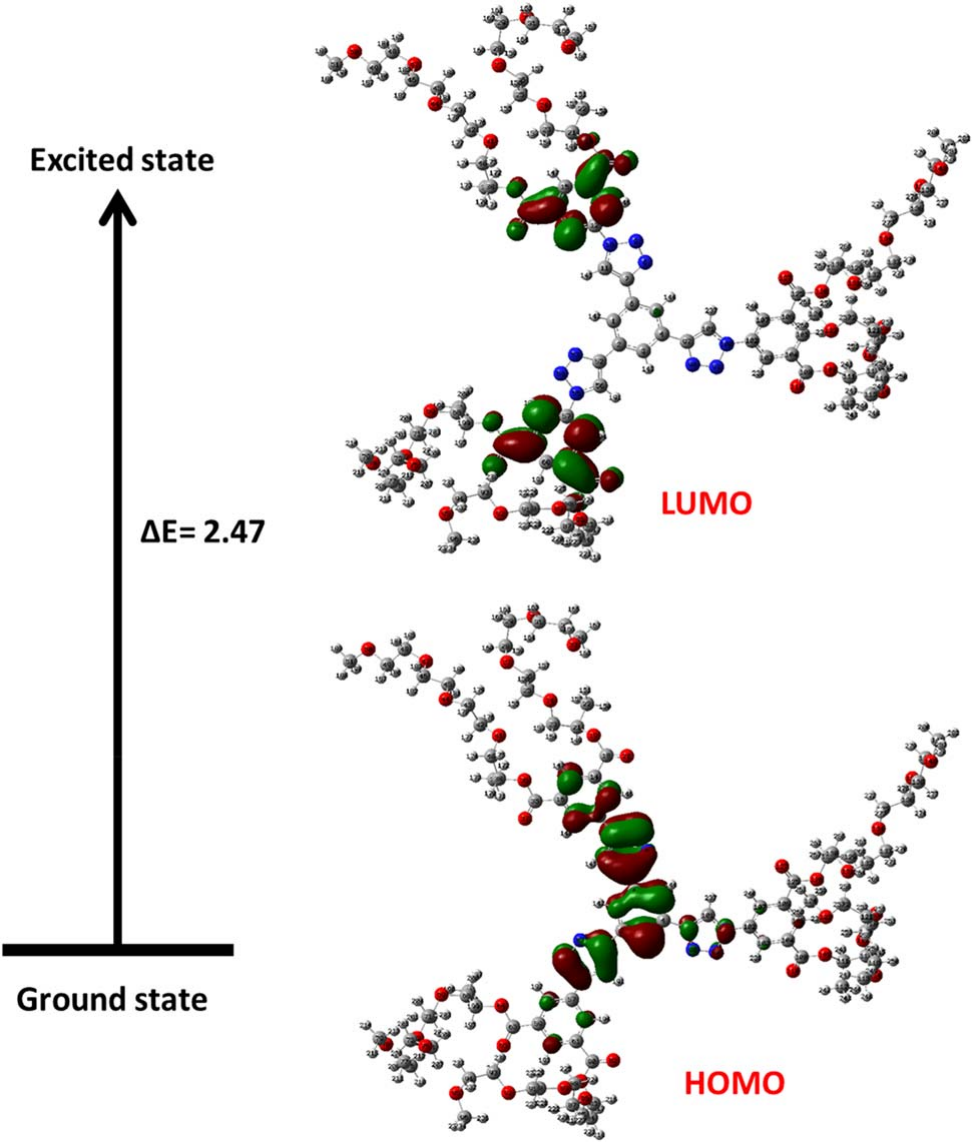
The HOMO and LUMO orbitals of dendrimer 9.

Frontier molecular orbitals are those that are necessary to understand kinetic predictability and chemical reactivity.^33^ Frontier molecular orbitals have a significant energy difference, which suggests weak reactivity and great chemical structural stability. In general, more energy is needed for the electrons to leave the stable level HOMO and enter the exciting level LUMO. The HOMO–LUMO gap (D), HOMO and LUMO energies, and all other chemical properties of dendrimer 9, the most active one, are shown in Table 3. Fig. 9 based on the computed global reactivity descriptors in Table 2, dendrimer 9 exhibits electronic properties indicative of a relatively stable yet moderately reactive chemical species. The fundamental HOMO–LUMO energy gap of 2.74 eV suggests a significant kinetic stability and low chemical reactivity, as it requires considerable energy to excite an electron. This is quantitatively supported by a chemical hardness (*η*) of 1.37 eV and a corresponding softness (*ζ*) of 0.36 eV^−1^, classifying it as a moderately hard molecule that is more likely to resist charge transfer. The calculated electronegativity (*χ*) of 6.69 eV and chemical potential (*µ*) of 3.41 eV point towards a tendency to attract electrons, albeit not overwhelmingly strong. Notably, the high electrophilicity index (*ω*) of 16.33 eV reveals a substantial capacity of the dendrimer to act as an electrophile, stabilizing itself by accepting electron density from a nucleophilic partner. This electrophilic character is further nuanced by the negative maximum charge transfer index (Δ*N*_max_ = −3.85), which implies that in an interaction with another species, the dendrimer would more readily act as an electron acceptor rather than a donor. In summary, these descriptors collectively characterize dendrimer 9 as a kinetically stable molecule with a pronounced inherent electrophilic reactivity (Fig. 9).

**Table 3:** Some global reactivity descriptors the computed for dendrimer 9

Parameters	Dendrimer 9
*E*_LUMO_	−0.19552
*E*_HOMO_	−0.29628
Energy band gap [*E*_HOMO_–*E*_LUMO_]eV	2.74
Ionization potential (*I* = −*E*_HOMO_)	8.06
Electron affinity (*A* = −*E*_LUMO_)	5.32
Chemical hardness (*η* = (*I* − *A*)/2)	1.37
Chemical softness (*ζ* = 1/2*η*)	0.36
Electronegativity (*χ* = (*I* + *A*)/2)	6.69
Chemical potential (*µ* = −(*I* + *A*)/2)	3.41
Electrophilicity index (*w* = *µ*^2^/2*η*)	16.33
Maximum charge transfer index (Δ*N*_max_ = −*µ*/*η*)	−3.85

#### 2.3.10. Molecular electrostatic potential (MEP) analysis

**Fig. 10:**
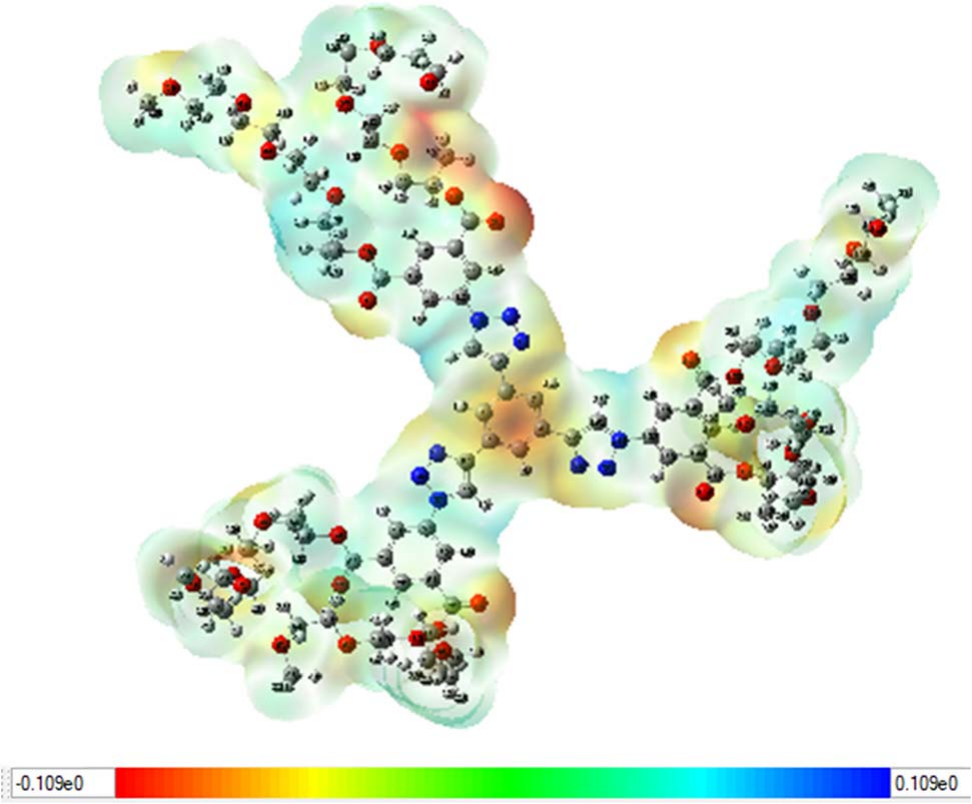
MESP map of dendrimer 9.

By identifying ligands or protein binding sites with the molecular electrostatic potential, or MESP, a favorable location for an electrophilic or nucleophilic attack can be determined.^34^ It can be used to determine the distribution of total charges positive and negative on the surface of a single molecule.^35^ Dendrimer 9's MESP was ascertained by geometry optimization with the B3LYP/3-21G basis set, as illustrated in Fig. 9. MESP is important because it can simultaneously represent, in terms of color grading, a molecule's size, structure, and positive, negative, and neutral electrostatic potential regions. This is helpful for studying the relationships between molecules and their physicochemical properties as well as molecular architectures.^36^ The red color displays the maximum negative area, which shows favorable sites for electrophilic attack, the blue color indicates the maximum positive area favorable for the nucleophilic attack, and the green color represents zero potential areas (Fig. 10).

## 3. Experimental

### 3.1. Chemistry

#### 3.1.1. Materials and methods

The chemicals and reagents used in these experiments are pure and were purchased from Sigma-Aldrich (Germany). We monitored the development of the chemical reactions by thin-layer chromatography (TLC). The ^1^H & ^13^C NMR Spectra were measured on Bruker's High-Performance Digital FT NMR spectrometer, Advance III (400 MHz for ^1^H and 100 MHz for ^13^C NMR). Shimadzu QP-2010 mass spectrometers plus (70 eV) were used to measure electron impact spectra. Mass spectrometry was conducted using the Thermo LTQ FT instrument (ESI, ESI-HRMS; mixtures of MeOH/H_2_O 75/25 with 0.5% formic acid) and the MSI Concept 1H (EI, 70 eV ionization), in addition to a QSTARXL Applied Biosystems ESI Q-TOF operating at an ISV of 950 V. MALDI-TOF MS spectra were obtained using the Bruker Autoflex3 Matrix Assisted Laser Desorption Ionization-Time of Flight Mass Spectrometer. Analytical UPLC measurements were performed with Waters Alliance systems consisting of a Waters Separations Module 2695, a Waters Diode Array detector 996 and a Waters Mass Detector ZQ 2000. (Mixtures and gradient mixtures of acetoneitrile/water, flow = 0.6 mL min^−1^) equipped with a 100 × 2.1 mm AQUITY HSST3 column (1.8 µm phenyl-hexyl material).

##### 3.1.1.1 Hexa(2,5,8,11-tetraoxatridecan-13-yl) 5,5′,5″-(benzene-1,3,5-triyltris(1*H*-1,2,3-triazole-4,1-diyl))triisophthalate (6)

A three necked flask was charged with 1,3,5-triethynylbenzene (1)^37^ (280 mg, 1.86 mmol, 1 equiv.) and di(2,5,8,11-tetraoxatridecan-13-yl) 5-azidoisophthalate (2)^38^ (3.61 g, 6.15 mmol, 3.3 equiv.), sodium ascorbate (111 mg, 0.56 mmol, 0.3 equiv.), TBTA (148 mg, 0.280 mmol, 0.15 equiv.) and a solvent mixture of H_2_O/^*tert*^BuOH/CH_2_Cl_2_ (1/2/8). The flask was evacuated and flushed with argon repeatedly (3 cycles). CuSO_4_·5H_2_O was added (70 mg, 0.28 mmol, 0.15 equiv.) and the mixture was stirred for 5 d at rt in the dark. After the acetylene starting material was consumed indicated by TLC monitoring (CH_2_Cl_2_/Acetone 5/5) the mixture was diluted with CH_2_Cl_2_ and transferred into a separation funnel. The organic phase was washed with aqueous Na_2_-EDTA solution (1×), the aqueous phase was extracted with CH_2_Cl_2_ (3×), and afterwards the combined organic phases were washed again with aqueous Na_2_-EDTA solution (2×) and once with aqueous sat. NaCl solution. After drying over MgSO_4_, filtration, and removal of the solvent *in vacuo* the title dendrimer was obtained by column chromatography (CH_2_Cl_2_/acetone 5/5) as yellow solid (83.3%). TLC (CH_2_Cl_2_/Acetone 5/5) *R*_f_ = 0.30. ^1^H-NMR (300 MHz, CDCl_3_): *δ* (ppm) = 8.81 (s, 3H, ArH), 8.75 (s, 9H, ArH), 8.56 (s, 3H, ArH), 4.58 (t, ^3^*J* = 4.5 Hz, 12H, CO_2_CH_2_), 3.91 (t, ^3^*J* = 4.92 Hz, 12H, CH_2_), 3.75–3.59 (m, 60H, CH_2_), 3.51–3.48 (m, 12H, CH_2_), 3.32 (s, 18H, OCH_3_). ^13^C-NMR (75 MHz, CDCl_3_): *δ* (ppm) = 164.50 (–CO_2_^–^), 147.91 (*C*_Ar_), 137.35 (*C*_Ar_), 132.50 (*C*_Ar_), 131.45 (*C*_Ar_), 130.50 (*C*_Ar_), 124.99 (*C*_Ar_), 122.90 (*C*_Ar_), 118.89 (*C*_Ar_), 71.88 (CH_2_), 70.72 (CH_2_), 70.64 (CH_2_), 70.57 (CH_2_), 70.46 (CH_2_), 69.00 (CH_2_), 65.00 (CH_2_), 58.96 (OCH_3_). UPLC *R*_t_ = 5.54 min, 99.75% peak area. HRMS (ESI) *m*/*z* = 1912.8628 (calcd 1912.8618 for [M + H^+^]).

##### 3.1.1.2 Tetra(2,5,8,11-tetraoxatridecan-13-yl) 5,5′-((5-ethynyl-1,3-phenylene)bis(1*H*-1,2,3-triazole-4,1-diyl))diisophthalate (3)

A three necked flask was charged with 1,3,5-triethynylbenzene (1)^37^ (1.50 mg, 10 mmol, 1 equiv.) and di(2,5,8,11-tetraoxatridecan-13-yl) 5-azidoisophthalate (2)^38^ (11.75 mg, 20 mmol, 2 equiv.), sodium ascorbate (396 mg, 2 mmol, 0.2 equiv.), TBTA (531 mg, 1 mmol, 0.1 equiv.) and a solvent mixture of H_2_O/^*tert*^BuOH/CH_2_Cl_2_ (1/2/8). The flask was evacuated and flushed with argon repeatedly (3 cycles). CuSO_4_·5H_2_O was added (250 mg, 1 mmol, 0.1 equiv.) and the mixture was stirred for 4 d at rt in the dark. After the acetylene starting material was consumed indicated by TLC monitoring (CH_2_Cl_2_/Acetone 5/5) the mixture was diluted with CH_2_Cl_2_ and transferred into a separation funnel. The organic phase was washed with aqueous Na_2_-EDTA solution (1×), the aqueous phase was extracted with CH_2_Cl_2_ (3×), and afterwards the combined organic phases were washed again with aqueous Na_2_-EDTA solution (2×) and once with aqueous sat. NaCl solution. After drying over MgSO_4_, filtration, and removal of the solvent *in vacuo* the title dendrimer was obtained by column chromatography(CH_2_Cl_2_/Acetone 5/5) as yellow oil (60%). TLC (CH_2_Cl_2_/Acetone 5/5) *R*_f_ = 0.48. ^1^H-NMR (300 MHz, CDCl_3_): *δ* (ppm) = 8.73 (s, 2H, ArH), 8.63 (s, 6H, ArH), 8.40–8.39 (m, 1H, ArH), 7.99 (ss, 2H, ArH), 4.51–4.48 (m, 8H, CO_2_CH_2_), 3.85–3.82 (m, 8H, CH_2_), 3.69–3.41 (m, 48H, CH_2_), 3.25 (s, 12H, OCH_3_), 3.21 (s, H, C≡CH). ^13^C-NMR (75 MHz, CDCl_3_): *δ* (ppm) = 164.38 (–CO_2_^–^), 147.29 (*C*_Ar_), 137.18 (*C*_Ar_), 134.70 (*C*_Ar_), 132.37 (*C*_Ar_), 130.90 (*C*_Ar_), 130.36 (*C*_Ar_), 128.99 (*C*_Ar_), 128.57 (*C*_Ar_), 127.90 (*C*_Ar_), 124.80 (*C*_Ar_), 123.60 (*C*_Ar_), 118.85 (*C*_Ar_), 82.71(C≡C), 78.62 (C≡CH), 72.50 (CHCH_2_), 71.80 (CH_2_), 70.62 (CH_2_), 70.56 (CH_2_), 70.50 (CH_2_), 70.39 (CH_2_), 70.24 (CH), 68.91 (CH_2_), 64.94 (CH_2_), 61.59 (CH), 58.87 (OCH_3_). UPLC *R*_t_ = 3.22, 99.9% peak area. HRMS (ESI) *m*/*z* = 1325.5957 (calcd 1325.5928 for [M + H^+^]).

##### 3.1.1.3 Tetra(2,5,8,11-tetraoxatridecan-13-yl) 5,5′-((5-(1-(3,5-bis((*R*)-3-methyl-2,5,8,11,14-pentaoxapentadecanoyl)phenyl)-1*H*-1,2,3-triazol-4-yl)-1,3-phenylene)bis(1*H*-1,2,3-triazole-4,1-diyl))diisophthalate (7)

A three necked flask was charged with tetra(2,5,8,11-tetraoxatridecan-13-yl) 5,5′-((5-ethynyl-1,3-phenylene)bis(1*H*-1,2,3-triazole-4,1-diyl))diisophthalate (3) (430 mg, 0.32 mmol, 1 equiv.) and di((*R*)-2,5,8,11-tetraoxatetradecan-13-yl) 5-azidoisophthalate (4)^26^ (260 mg, 0.42 mm ol, 1.3 equiv.), sodium ascorbate (6 mg, 0.03 mmol, 0.1 equiv.), TBTA (9 mg, 0.016 mmol, 0.05 equiv.) and a solvent mixture of H_2_O/^*tert*^BuOH/CH_2_Cl_2_ (1/2/8). The flask was evacuated and flushed with argon repeatedly (3 cycles). CuSO_4_·5H_2_O was added (4 mg, 0.016 mmol, 0.05 equiv.) and the mixture was stirred for 5 d at rt in the dark. After the acetylene starting material was consumed indicated by TLC monitoring (CH_2_Cl_2_/Acetone 5/5) the mixture was diluted with CH_2_Cl_2_ and transferred into a separation funnel. The organic phase was washed with aqueous Na_2_-EDTA solution (1×), the aqueous phase was extracted with CH_2_Cl_2_ (3×), and afterwards the combined organic phases were washed again with aqueous Na_2_-EDTA solution (2×) and once with aqueous sat. NaCl solution. After drying over MgSO_4_, filtration, and removal of the solvent *in vacuo* the title dendrimer was obtained by column chromatography (CH_2_Cl_2_/Acetone 5/5) as yellow oil (86.5%). TLC (CH_2_Cl_2_/Acetone 5/5) *R*_f_ = 0.40. ^1^H-NMR (300 MHz, CDCl_3_): *δ* (ppm) = 8.79–8.71 (m, 12H, ArH), 8.57–8.55 (m, 3H, ArH), 5.46–5.40 (m, 2H, CO_2_CH), 4.59–4.56 (m, 8H, CO_2_CH_2_), 3.92–3.89 (m, 8H, CH_2_), 3.75–3.59 (m, 64H, CH_2_), 3.51–3.48 (m, 12H, CH_2_), 3.32 (ss, 18H, OCH_3_), 1.44 (d, ^2^*J* = 6.47 Hz, 6H, CHCH_3_). ^13^C-NMR (75 MHz, CDCl_3_): *δ* (ppm) = 164.48 (–CO_2_^–^), 164.05 (–CO_2_^–^), 147.95 (*C*_Ar_), 137.33 (*C*_Ar_), 137.27 (*C*_Ar_), 132.93 (*C*_Ar_), 132.53 (*C*_Ar_), 131.45 (*C*_Ar_), 130.59 (*C*_Ar_), 125.08 (*C*_Ar_), 124.96 (*C*_Ar_), 122.99 (*C*_Ar_), 118.77 (*C*_Ar_), 73.60 (CH_2_), 71.86 (CH_2_), 71.31 (CH_2_), 70.82 (CH_2_), 70.68 (CH_2_), 70.62 (CH_2_), 70.56 (CH_2_), 70.46 (CH_2_), 68.97 (CH_2_), 64.99 (CH_2_), 58.96 (OCH_3_), 16.81 (CHCH_3_). UPLC *R*_t_ = 4.0, 100. % peak area. HRMS (ESI) *m*/*z* = 1940.8979 (calcd 1940.8931 for [M + H^+^]).

##### 3.1.1.4 Tetra((*R*)-2,5,8,11-tetraoxatetradecan-13-yl) 5,5′-((5-ethynyl-1,3-phenylene)bis(1*H*-1,2,3-triazole-4,1-diyl))diisophthalate (5)

A three necked flask was charged with 1,3,5-triethynylbenzene (1)^37^ (1.50 g, 10 mmol, 1 equiv.) and di((*R*)-2,5,8,11-tetraoxatetradecan-13-yl) 5-azidoisophthalate (4)^26^ (12.31 mg, 20 mmol, 2 equiv.), sodium ascorbate (396 mg, 2 mmol, 0.2 equiv.), TBTA (531 mg, 1 mmol, 0.1 equiv.) and a solvent mixture of H_2_O/^*tert*^BuOH/CH_2_Cl_2_ (1/2/8). The flask was evacuated and flushed with argon repeatedly (3 cycles) CuSO_4_·5H_2_O was added (250 mg, 1 mmol, 0.1 equiv.) and the mixture was stirred for 3 d at rt in the dark. After the acetylene starting material was consumed indicated by TLC monitoring (CH_2_Cl_2_/Acetone 7/3) the mixture was diluted with CH_2_Cl_2_ and transferred into a separation funnel. The organic phase was washed with aqueous Na_2_-EDTA solution (1×), the aqueous phase was extracted with CH_2_Cl_2_ (3×), and afterwards the combined organic phases were washed again with aqueous Na_2_-EDTA solution (2×) and once with aqueous sat. NaCl solution. After drying over MgSO_4_, filtration, and removal of the solvent *in vacuo* the title dendrimer was obtained by column chromatography (CH_2_Cl_2_/Acetone 7/3) as yellow oil (64%). TLC (CH_2_Cl_2_/Acetone 7/3) *R*_f_ = 0.58. ^1^H-NMR (300 MHz, CDCl_3_): *δ* (ppm) = 8.71 (s, 2H, ArH), 8.59 (s, 6H, ArH), 8.39 (t, ^3^*J* = 2.97 Hz, H, ArH), 7.97 (d, 2H, ArH), 5.37–5.27 (m, 4H, CO_2_CH), 3.72–3.49 (m, 56H, CH_2_), 5.37–5.27 (m, 4H, CO_2_CH), 3.22 (s, 12H, OCH_3_), 3.20 (s, H, C≡CH), 1.34 (d, ^2^*J* = 6.47 Hz, 12H, CHCH_3_),. ^13^C-NMR (75 MHz, CDCl_3_): *δ* (ppm) = 163.92 (–CO_2_^–^), 147.28 (*C*_Ar_), 137.13 (*C*_Ar_), 130.92 (*C*_Ar_), 130.32 (*C*_Ar_), 128.94 (*C*_Ar_), 124.71 (*C*_Ar_), 123.57 (*C*_Ar_), 123.19 (*C*_Ar_), 118.90 (*C*_Ar_), 82.71(C≡C)), 78.63 (C≡CH), 73.49 (CHCH_2_), 71.77 (CH_2_), 71.22 (CH_2_), 70.75 (CH_2_), 70.53 (CH_2_), 70.47 (CH_2_), 70.36 (CH), 58.83 (OCH_3_), 16.70 (CHCH_3_), UPLC *R*_t_ = 3.05, 98. % peak area. HRMS (ESI) *m*/*z* = 1381.6534 (calcd 1381.6554 for [M + H^+^]).

##### 3.1.1.5 Tetra((*R*)-2,5,8,11-tetraoxatetradecan-13-yl) 5,5′-((5-(1-(3,5-di(2,5,8,11,14-pentaoxapentadecanoyl)phenyl)-1*H*-1,2,3-triazol-4-yl)-1,3-phenylene)bis(1*H*-1,2,3-triazole-4,1-diyl))diisophthalate (8)

A three necked flask was charged with tetra((*R*)-2,5,8,11-tetraoxatetradecan-13-yl) 5,5′-((5-ethynyl-1,3-phenylene)bis(1*H*-1,2,3-triazole-4,1-diyl))diisophthalate (5) (540 mg, 0.39 mmol, 1 equiv.) and di(2,5,8,11-tetraoxatridecan-13-yl) 5-azidoisophthalate (2)^38^ (299 mg, 0.50 mmol, 1.3 equiv.), sodium ascorbate (8 mg, 0.03 mmol, 0.1 equiv.), TBTA (10 mg, 0.02 mmol, 0.05 equiv.) and a solvent mixture of H_2_O/^*tert*^BuOH/CH_2_Cl_2_ (1/2/8). The flask was evacuated and flushed with argon repeatedly (3 cycles). CuSO_4_·5H_2_O was added (5 mg, 0.02 mmol, 0.05 equiv.) and the mixture was stirred for 5 d at rt in the dark. After the acetylene starting material was consumed indicated by TLC monitoring (CH_2_Cl_2_/cetone 7/3) the mixture was diluted with CH_2_Cl_2_ and transferred into a separation funnel. The organic phase was washed with aqueous Na_2_-EDTA solution (1×), the aqueous phase was extracted with CH_2_Cl_2_ (3×), and afterwards the combined organic phases were washed again with aqueous Na_2_-EDTA solution (2×) and once with aqueous sat. NaCl solution. After drying over MgSO_4_, filtration, and removal of the solvent *in vacuo* the title dendrimer was obtained by column chromatography (CH_2_Cl_2_/Acetone 6/4) as yellow oil (93.4%). TLC (CH_2_Cl_2_/Acetone 6/4) *R*_f_ = 0.45. ^1^H-NMR (300 MHz, CDCl_3_): *δ* (ppm) = 8.87 (s, 3H, ArH), 8.59 (s, 6H, ArH), 8.49–8.46 (ss, 3H, ArH), 8.35–8.33 (ss, 3H, ArH), 5.30–5.24 (m, 4H, CO_2_CH), 4.41 (s, 4H, CO_2_CH_2_), 3.79–3.31 (m, 84H, CH_2_), 3.16–3.14 (ss, 18H, OCH_3_), 1.30 (d, ^2^*J* = 6.37 Hz, 12H, CHCH_3_). ^13^C-NMR (75 MHz, CDCl_3_): *δ* (ppm) = 164.29 (–CO_2_^–^), 163.90 (–CO_2_^–^), 147.59 (*C*_Ar_), 137.21 (*C*_Ar_), 132.60 (*C*_Ar_), 132.15 (*C*_Ar_), 131.26 (*C*_Ar_), 129.99 (*C*_Ar_), 128.89 (*C*_Ar_), 128.47 (*C*_Ar_), 127.80 (*C*_Ar_), 124.47 (*C*_Ar_), 122.41 (*C*_Ar_), 119.15 (*C*_Ar_), 73.49 (CH_2_), 71.70 (CH_2_), 71.16 (CH_2_), 70.74 (CH_2_), 70.58 (CH_2_), 70.50 (CH_2_), 70.47 (CH_2_), 70.40 (CH_2_), 70.28 (CH_2_), 68.84 (CH_2_), 64.84 (CH_2_), 58.75 (OCH_3_), 16.69 (CHCH_3_). UPLC *R*_t_ = 3.96, 100. % peak area. HRMS (ESI) *m*/*z* = 1968.9292 (calcd 1968.9244 for [M + H^+^]).

### 3.2. Cytotoxicity assay

#### 3.2.1. Materials

The chemicals utilized in this study comprised RPMI-1640 medium, MTT, and DMSO, all sourced from Sigma Co., located in St. Louis, USA. The cell lines involved were colorectal carcinoma (HCT-116), hepatocellular carcinoma (HepG-2), mammary gland-breast cancer (MCF-7), and human lung fibroblast (WI38), all procured from ATCC through VACSERA in Cairo, Egypt.

#### 3.2.2. Procedure

A study was carried out to evaluate the inhibitory effects of dendrimers 6–9 and 12 on the growth of cancer cell lines through the MTT assay. This colorimetric assay is based on mitochondrial succinate dehydrogenase, which converts yellow MTT into a purple formazan in viable cells.^39,40^ The cancer cell lines were cultivated in RPMI-1640 medium that was supplemented with 10% fetal bovine serum, penicillin (100 units per mL), and streptomycin (100 µg mL^−1^) at a temperature of 37 °C with 5% CO_2_. Afterward, the cells were plated in a 96-well plate at a density of 1.0 × 10^4^ cells per well and incubated for two days prior to treatment with the dendrimers at various concentrations for a period of 48 hours. Upon completion of the treatment, 20 µL of MTT solution (5 mg mL^−1^) was added, and the cells were incubated for an additional 4 hours. To dissolve the formazan, 100 µL of DMSO was introduced, and the colorimetric assay was conducted at an absorbance of 570 nm utilizing the EXL 800 plate reader (USA). The relative cell viability (%) was determined using the following formula: cell viability (%) = A at 570 nm of treated samples/A at 570 nm of untreated samples ×100.

#### 3.2.3. Statistical analysis

The statistical analysis was conducted using SPSS Statistics for Windows (version 20.0, SPSS, Chicago, IL, USA). Duncan's test was used to identify any significant differences between the treatments and controls, in addition to those among the treatments themselves. The threshold for significance in this analysis was set at 0.05.

### 3.3. System preparation and molecular docking

The 3D X-ray derived structure of Estrogen Receptor Alpha (ERα) in complex with 4-hydroxytamoxifen was retrieved from the protein data bank with code 3ERT^41^ and prepared using UCSF Chimera.^42^ Using PROPKA, pH was fixed and optimized to 7.5.^43^ The synthesized 2D structure was drawn using ChemBioDraw Ultra 12.1.^44^ The steepest descent approach and MMFF94 force field in Avogadro software^45^ were used to optimise 2D structure for energy minimization. In preparation for molecular dynamic, hydrogen atoms were removed using UCSF chimaera.^42^

#### 3.3.1. Molecular docking

AutoDock Vina was used for docking calculations,^46^ and Gasteiger partial charges^47^ were allocated during docking. The AutoDock graphical user interface offered by MGL tools was used to outline the AutoDock atom types.^48^ The *x*, *y*, *z* AutoDock Vina grid center coordinates used are 16.92 Å, 4.13 Å, 0.66 Å and the size of the search space were set to 31.37 Å × 34.98 Å × 29.62 Å and exhaustiveness = 8. The Lamarckian genetic algorithm^49^ was used to create docked conformations in descending order based on their docking energy.

#### 3.3.2. Molecular dynamic (MD) simulations

Molecular dynamics (MD) simulations provide a powerful means to investigate the physical motions of atoms and molecules within biological systems, offering insights into dynamic processes such as conformational changes and molecular associations that are difficult to access experimentally.^50^ In this study, all MD simulations were performed using the GPU-accelerated PMEMD engine in the AMBER 18 software package.^51^ Prior to simulation, partial atomic charges for each ligand were assigned using the General Amber Force Field (GAFF) within the ANTECHAMBER utility.^52^ Each system was then prepared with the LEaP module of AMBER 18. The protein-ligand complex was solvated in an orthorhombic box of TIP3P water molecules, extending 10 Å from the solute surface. Systems were neutralized by adding Na^+^ and Cl^−^ counterions. The energy of each system was minimized in two stages: first, a restrained minimization of 2000 steps with a 500 kcal mol^−1^ Å^−2^ restraint on solute atoms, followed by a 1000-step unrestrained minimization using the conjugate gradient algorithm. Following minimization, systems were gradually heated from 0 K to 300 K over 500 ps under constant volume (NVT) conditions, with a 10 kcal mol^−1^ Å^−2^ restraint on solute atoms and a Langevin thermostat collision frequency of 1 ps^−1^. This was followed by a 500 ps equilibration at 300 K under constant pressure (NPT) conditions, maintained at 1 bar using the Berendsen barostat.^53^ Production simulations were conducted for 45 ns in the NPT ensemble at 300 K and 1 bar, using the Langevin thermostat (collision frequency = 1 ps^−1^) and Berendsen barostat (pressure relaxation time = 2 ps). The SHAKE algorithm was applied to constrain bonds involving hydrogen, permitting a 2 fs integration time step. All simulations employed the SPFP precision model and used randomized seeding. It worth to noted that the residue numbers in MD analyses refer to the topology file generated by AMBER's tleap and may differ from the canonical PDB numbering of 3ERT.

#### 3.3.3. Post-MD analysis

After saving the trajectories obtained by MD simulations every 1 ps, the trajectories were analyzed using the AMBER18 suite's CPPTRAJ^54^ module. The origin data analysis program and Chimera^42^ were used to create all graphs and visualizations.

#### 3.3.4. Thermodynamic calculation

The Poisson-Boltzmann or generalized born and surface area continuum solvation (MM/PBSA and MM/GBSA) approach has been found to be useful in the estimation of ligand-binding affinities.^55^ The protein–ligand complex molecular simulations used by MM/GBSA and MM/PBSA compute rigorous statistical-mechanical binding free energy within a defined force field. Binding free energy averaged over 450 snapshots extracted from the entire 45 ns trajectory. The estimation of the change in binding free energy (Δ*G*) for each molecular species (complex, ligand, and receptor) can be represented as follows:^56^

1 Δ*G*_bind_ = *G*_complex_ − *G*_receptor_ − *G*_ligand_

2 Δ*G*_bind_ = *E*_gas_ + *G*_sol_ − TS

3 *E*_gas_ = *E*_int_ + *E*_vdw_ + *E*_ele_

4 *G*_sol_ = *G*_GB_ + *G*_SA_

5 *G*_SA_ = *γ*SASA

The terms *E*_gas_, *E*_int_, *E*_ele_, and *E*_vdw_ symbolize the gas-phase energy, internal energy, Coulomb energy, and van der Waals energy. The *E*_gas_ was directly assessed from the FF14SB force field terms. Solvation-free energy (*G*_sol_) was evaluated from the energy involvement from the polar states (GGB) and non-polar states (*G*). The non-polar solvation free energy (GSA) was determined from the Solvent Accessible Surface Area (SASA)^57^ using a water probe radius of 1.4 Å. In contrast, solving the GB equation assessed the polar solvation (GGB) contribution. Items *S* and *T* symbolize the total entropy of the solute and temperature, respectively. The MM/GBSA-binding free energy method in Amber18 was used to calculate the contribution of each residue to the total binding free energy.

#### 3.3.5. DCCM analysis

We used dynamic cross-correlation analysis to investigate the fluctuations and movements in the backbone of the α carbon atoms.^58^ The cross-correlation elements *C*_*ij*_ between Cα atoms of residues *i* and *j* of proteins can be computed based on structural extracted from MD trajectories using the following equations:^59^6
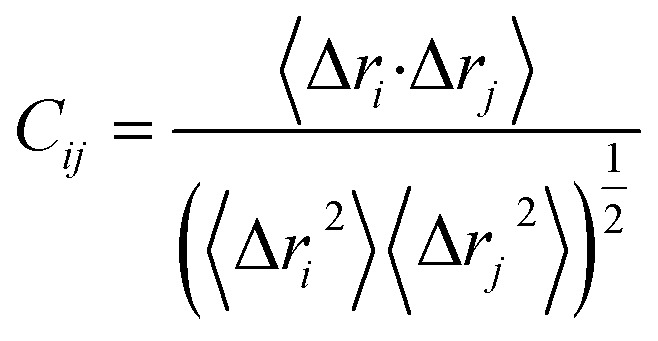
where Δ*r*_*i*_ is the displacement of the *i*th Cα atom relative to its averaged position. The Δ*r*_*i*_ is the displacement of the *i*th Cα atom relative to its averaged position. Significantly correlated movements are symbolized by *C*_*ij*_ = 1, while *C*_*ij*_ = −1 symbolized highly anticorrelated movements in the trajectory. The divergence of motion from 1 and −1 indicates that *i* and *j* movements are anticorrelated. The DCCM matrix was carried out using the CPPTRAJ package in Amber 18, and the matrices were plotted and evaluated using Origin software (https://www.originlab.com/).

## 4. Conclusion

A modular CuAAC click-chemistry approach enabled the efficient synthesis of chiral dendrimers with precisely controlled stereochemistry and generation. Systematic biological evaluation revealed a clear structure-activity relationship, identifying the fully chiral first-generation dendrimer (compound 9) as the most potent and selective anticancer candidate. Compound 9 exhibited strong cytotoxicity against HCT-116, HepG-2, and MCF-7 cells, coupled with significantly reduced toxicity toward normal WI-38 fibroblasts. In contrast, the second-generation dendrimer showed diminished activity, suggesting that increased molecular size may hinder cellular internalization. It should be noted that cellular uptake and detailed physicochemical properties were not directly measured here; these remain important subjects for future validation studies. Molecular dynamics and MM/GBSA analyses qualitatively confirmed stable and favorable binding of compound 9 to ERα, predominantly driven by hydrophobic interactions, providing a plausible mechanistic basis for its activity in ERα-positive models such as MCF-7 cells. It should be noted that the MM/GBSA-derived binding energies are presented for comparative and mechanistic insight, and the absolute values may not fully capture entropic contributions, particularly for large dendritic systems. The compound's efficacy in other cell lines (HCT-116, HepG-2) suggests that additional, non-receptor-mediated mechanisms may also contribute to its broad-spectrum cytotoxicity, warranting further investigation into alternative targets and pathways. Future efforts to enhance the therapeutic potential of this class of chiral dendrimers should focus on improving cellular uptake and tumor selectivity. Surface modification *via* PEGylation or conjugation with cell-penetrating peptides (*e.g.*, TAT, RGD) could enhance solubility, reduce non-specific interactions, and promote receptor-mediated endocytosis. Generation and size optimization such as designing intermediate generation variants (*e.g.*, G1.5–G2.5) may better balance multivalency with cellular permeability, addressing the uptake limitation observed with the larger G2 dendrimer 12. Incorporating stimuli-responsive linkages (*e.g.*, pH- or enzyme-cleavable bonds) within the dendritic framework could enable controlled drug release in the tumor microenvironment. Furthermore, attaching tumor-specific targeting ligands (*e.g.*, folic acid, HER2 antibodies) would improve selectivity and internalization in cancer cells. These strategic modifications, building upon the chiral click-assembly platform established here, could translate these promising *in vitro* leads into more effective and targeted *in vivo* therapeutics.

## Conflicts of interest

No potential conflict of interest was reported by the author.

## Supplementary Material

RA-016-D6RA00668J-s001

## Data Availability

The data supporting this article have been included as part of the supplementary information (SI). Supplementary information is available. See DOI: https://doi.org/10.1039/d6ra00668j.
