# Supplementary material for: Rational design of click-assembled chiral dendrimers: anticancer activity and molecular dynamics study
Source: RSC Adv. 2026 Mar 2;16(13):11763–78. doi: 10.1039/d6ra00668j (PMC12951598; doi:10.1039/d6ra00668j)
Supplement: RA-016-D6RA00668J-s001 [file RA-016-D6RA00668J-s001.pdf]

Supplementary Information (SI) for RSC Advances.
This journal is © The Royal Society of Chemistry 2026

# Rational Design of Click-Assembled Chiral Dendrimers: Anticancer Activity and Molecular Dynamics Study

Tamer El Malah[a] * and Ahmed A. El-Rashedy[b,c]

[a] *Photochemistry Department, Chemical Industries Research Institute, National Research Centre, 33 El Buhouth Street, P.O. Box 12622, Cairo, Egypt*

[b] *Chemistry of Natural and Microbial Products Department, National Research Centre, Dokki, 12622 Cairo, Egypt*

[c] *Department of Organic and Medicinal Chemistry, Faculty of Pharmacy, University of Sadat City, Menoufia 32897, Egypt*

*Corresponding author: tmara_nrc3000@yahoo.com (Tamer El Malah)

# Table of Contents

**Figure S1.** $^{1}H$ NMR spectrum of compound **6** (400 MHz, $CDCl_3$, 25 °C)……………………... **3**

**Figure S2.** $^{13}C$ NMR spectrum of compound **6** (100 MHz, CDCl3, 25 °C)…………………….. **3**

**Figure S3.** $^{1}H$ NMR spectrum of compound **3** (400 MHz, $CDCl_3$, 25 °C)………………………. **4**

**Figure S4.** $^{13}C$ NMR spectrum of compound **3** (100 MHz, $CDCl_3$, 25 °C)…………………….. **4**

**Figure S5.** $^{1}H$ NMR spectrum of compound **7** (100 MHz, $CDCl_3$, 25 °C)………………………... **5**

**Figure S6.** $^{13}C$ NMR spectrum of compound **7** (400 MHz, $CDCl_3$, 25 °C)……………………….. **5**

**Figure S7.** $^{1}H$ NMR spectrum of compound **5** (400 MHz, $CDCl_3$, 25 °C)………………………... **6**

**Figure S8.** $^{13}C$ NMR spectrum of compound **5** (100 MHz, $CDCl_3$, 25 °C)……………………….. **6**

**Figure S9.** $^{1}H$ NMR spectrum of compound **8** (400 MHz, $CDCl_3$, 25 °C)………………………... **7**

**Figure S10.** $^{13}C$ NMR spectrum of compound **8** (100 MHz, $CDCl_3$, 25 °C)…………………… **7**

**Table S1.** Per-residue binding free energy contributions (kcal/mol) of key amino acids to the binding of dendrimer 9 with the ERα receptor (AMBER system numbering). **8**

**Compound 6**

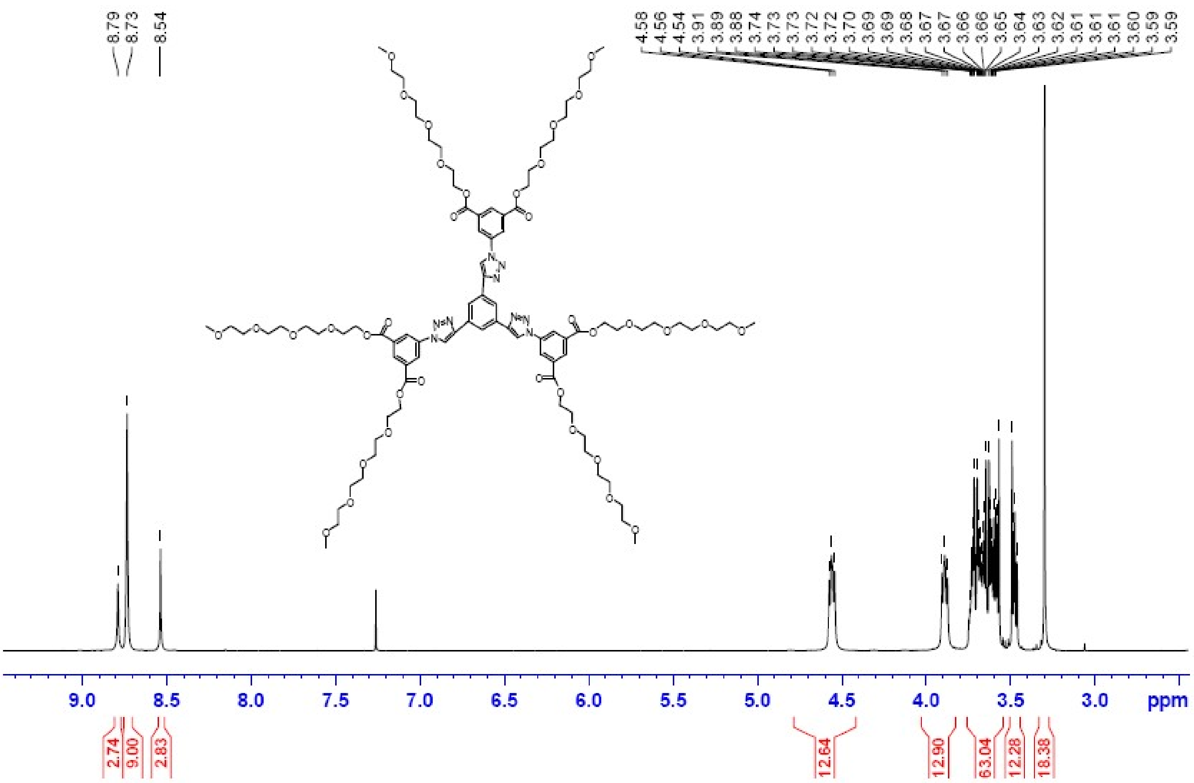

**Figure S1.** $^{1}$H NMR spectrum of compound **6** (400 MHz, $CDCl_3$, 25 °C)

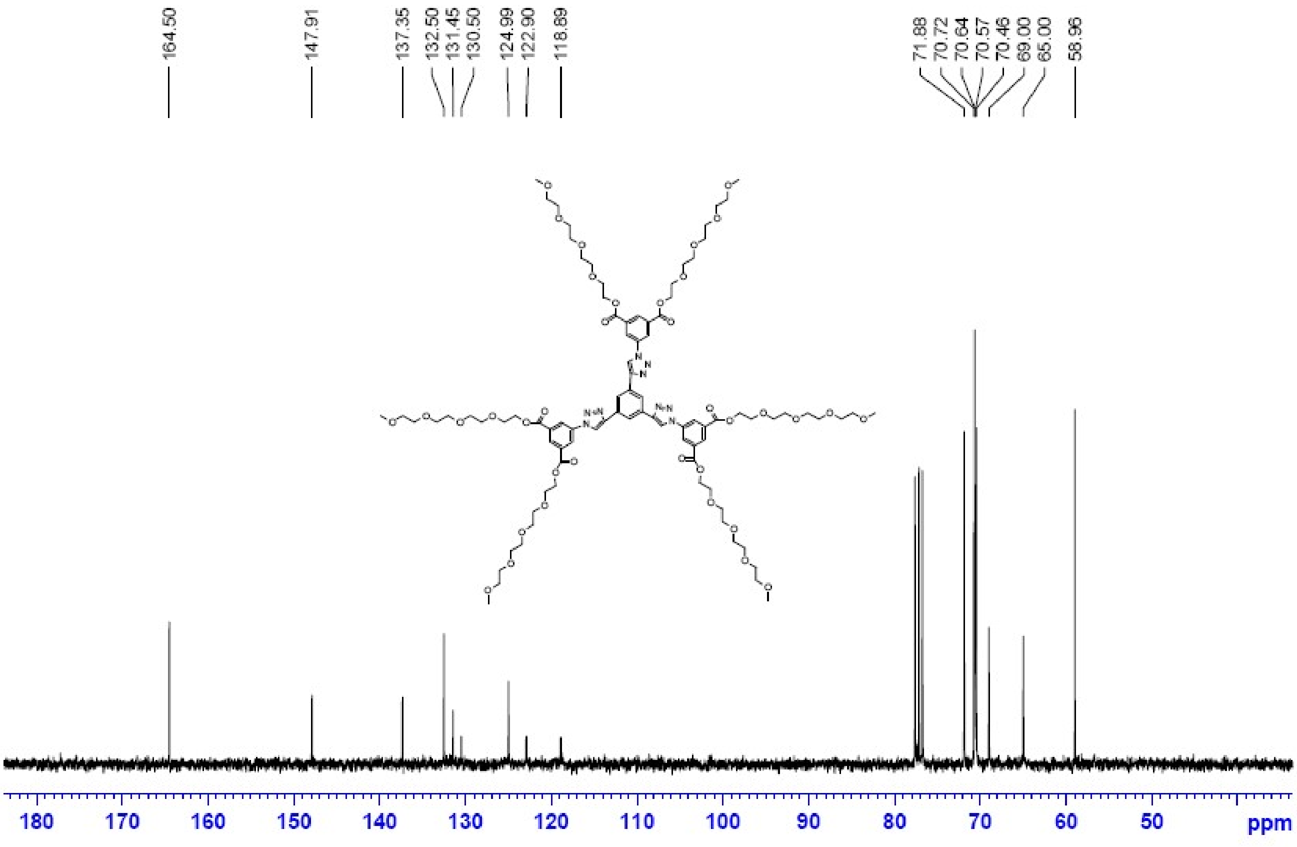

**Figure S2.** $^{13}$C NMR spectrum of compound **6** (100 MHz, $CDCl_3$, 25 °C)

**Compound 3**

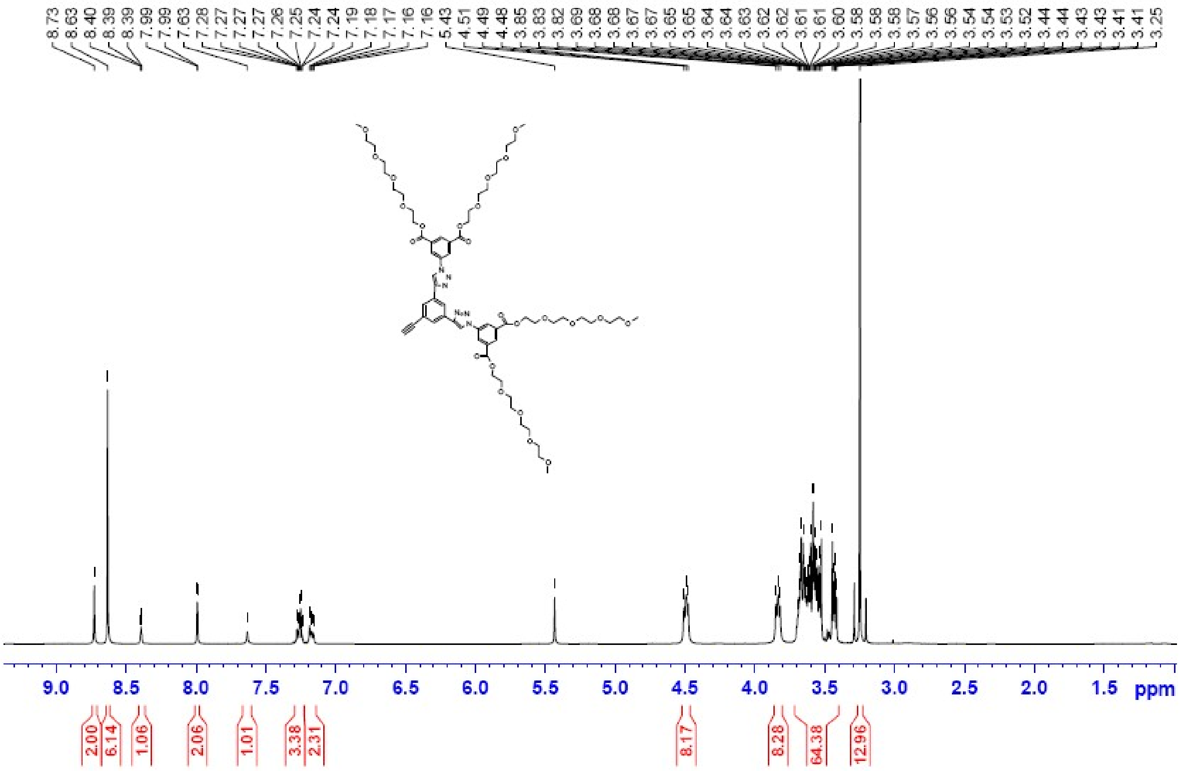

**Figure S3.** $^{1}H$ NMR spectrum of compound **3** (400 MHz, $CDCl_3$, 25 °C)

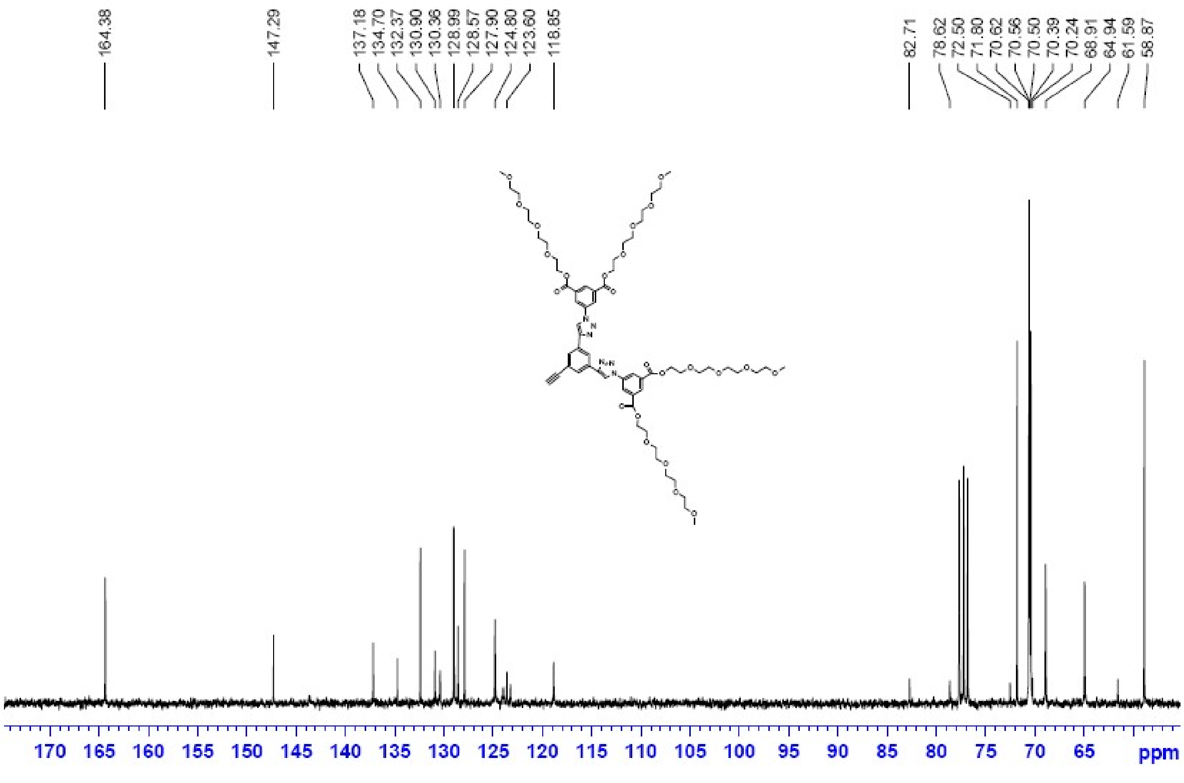

**Figure S4.** $^{13}C$ NMR spectrum of compound **3** (100 MHz, $CDCl_3$, 25 °C)

## Compound 7

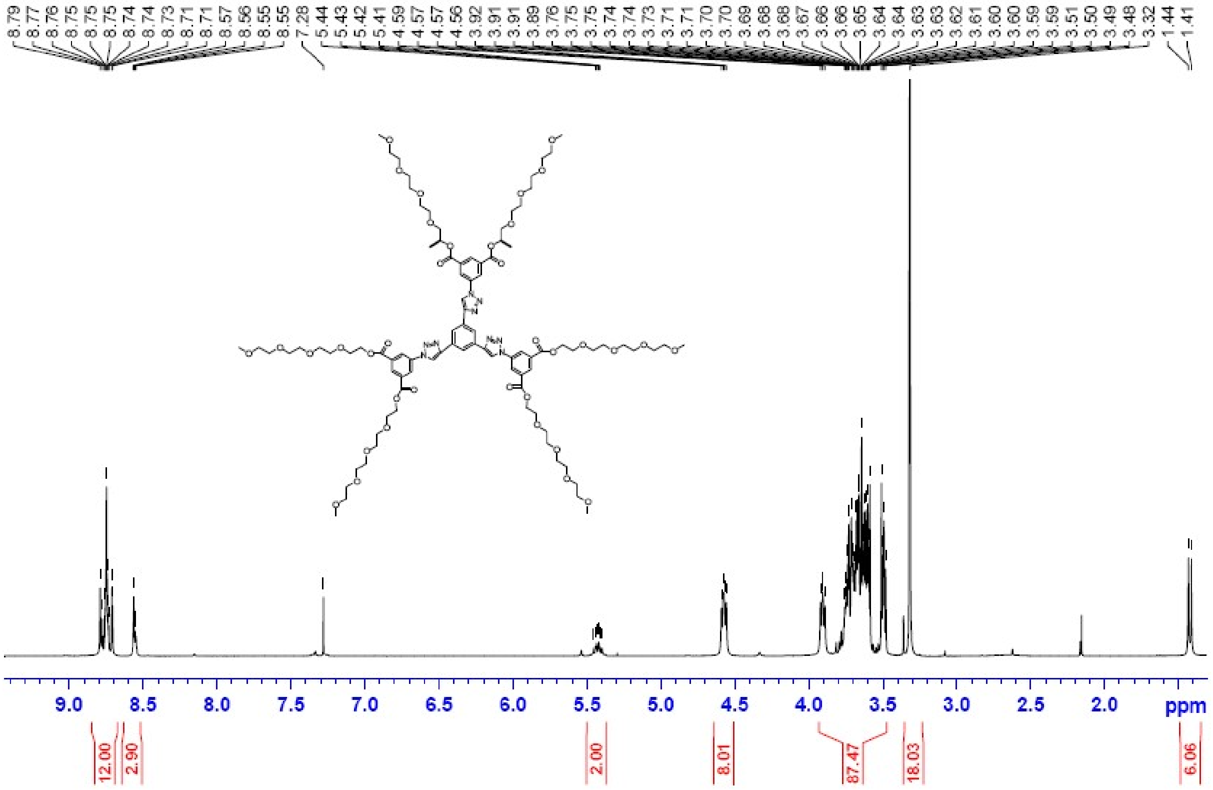


**Figure S5.** $^{1}H$ NMR spectrum of compound **7** (400 MHz, $CDCl_3$, 25 °C)

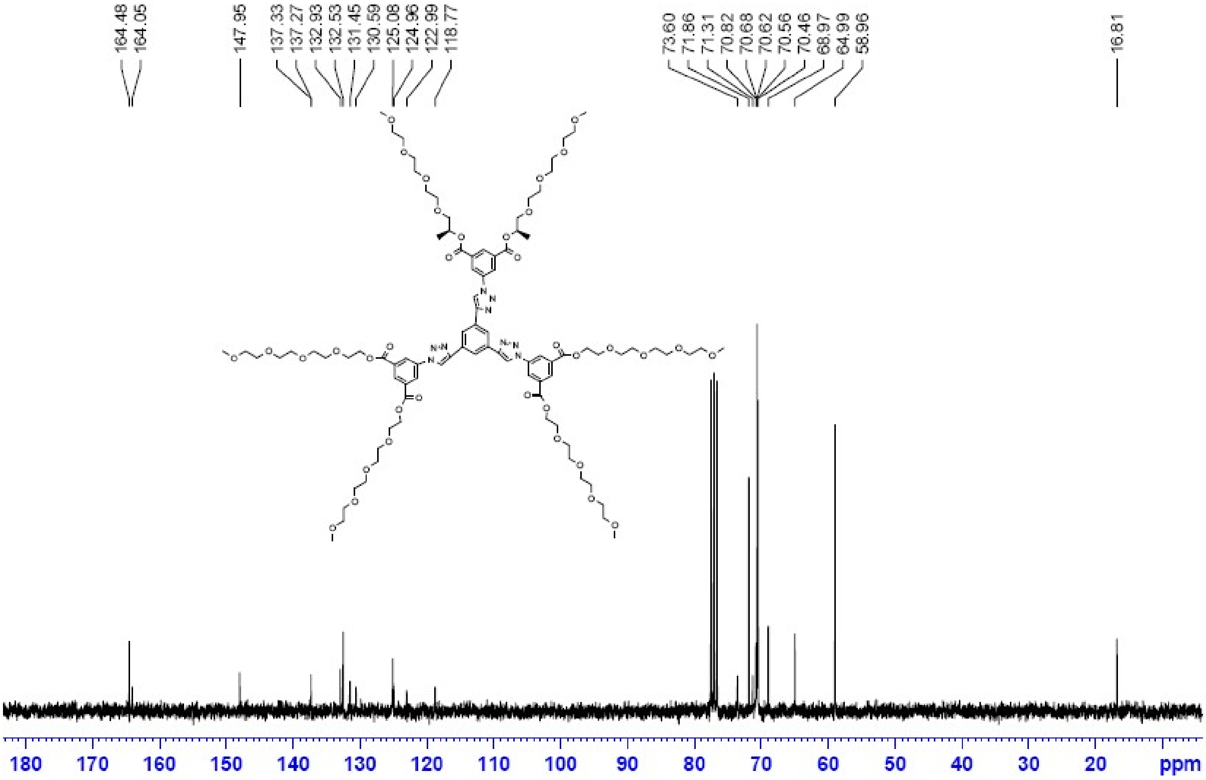


**Figure S6.** $^{13}C$ NMR spectrum of compound **7** (100 MHz, $CDCl_3$, 25 °C)

# Compound 5

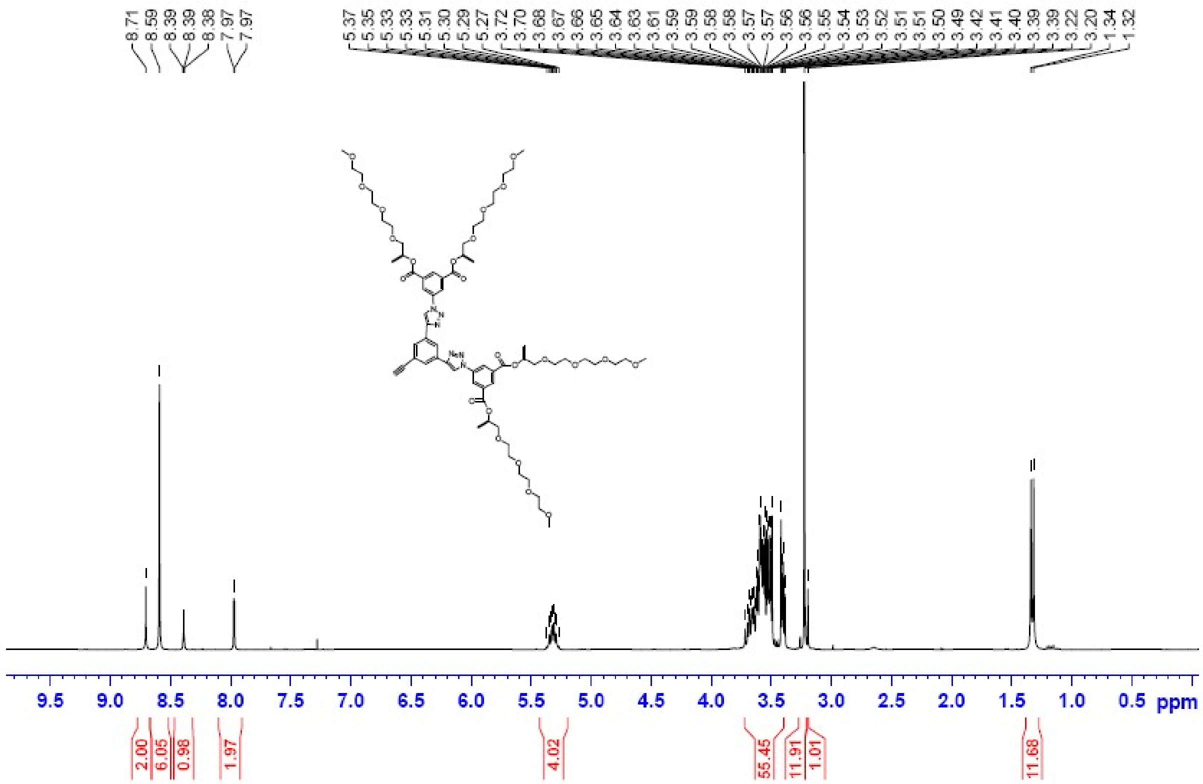


**Figure S7.** $^{1}$H NMR spectrum of compound **5** (400 MHz, $CDCl_3$, 25 °C)

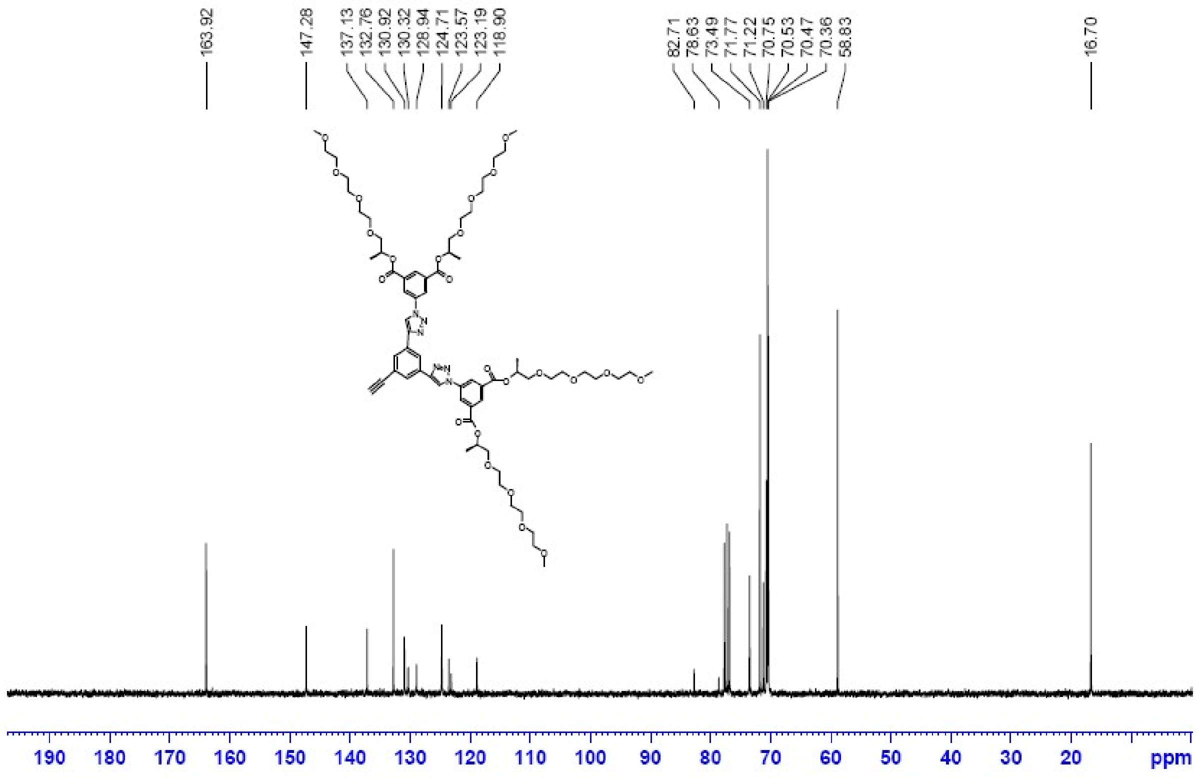


**Figure S8.** $^{13}$C NMR spectrum of compound **5** (100 MHz, $CDCl_3$, 25 °C)

## Compound 8

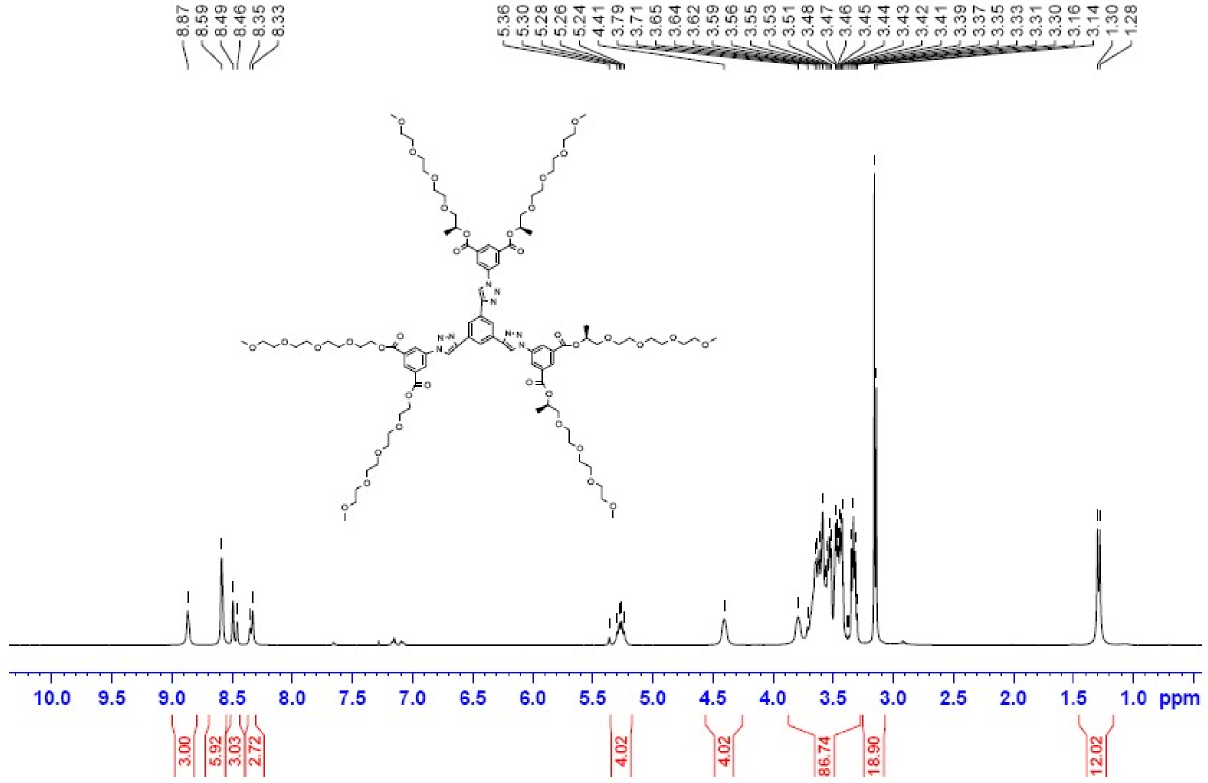


**Figure S9.** $^{1}H$ NMR spectrum of compound **8** (400 MHz, $CDCl_3$, 25 °C)

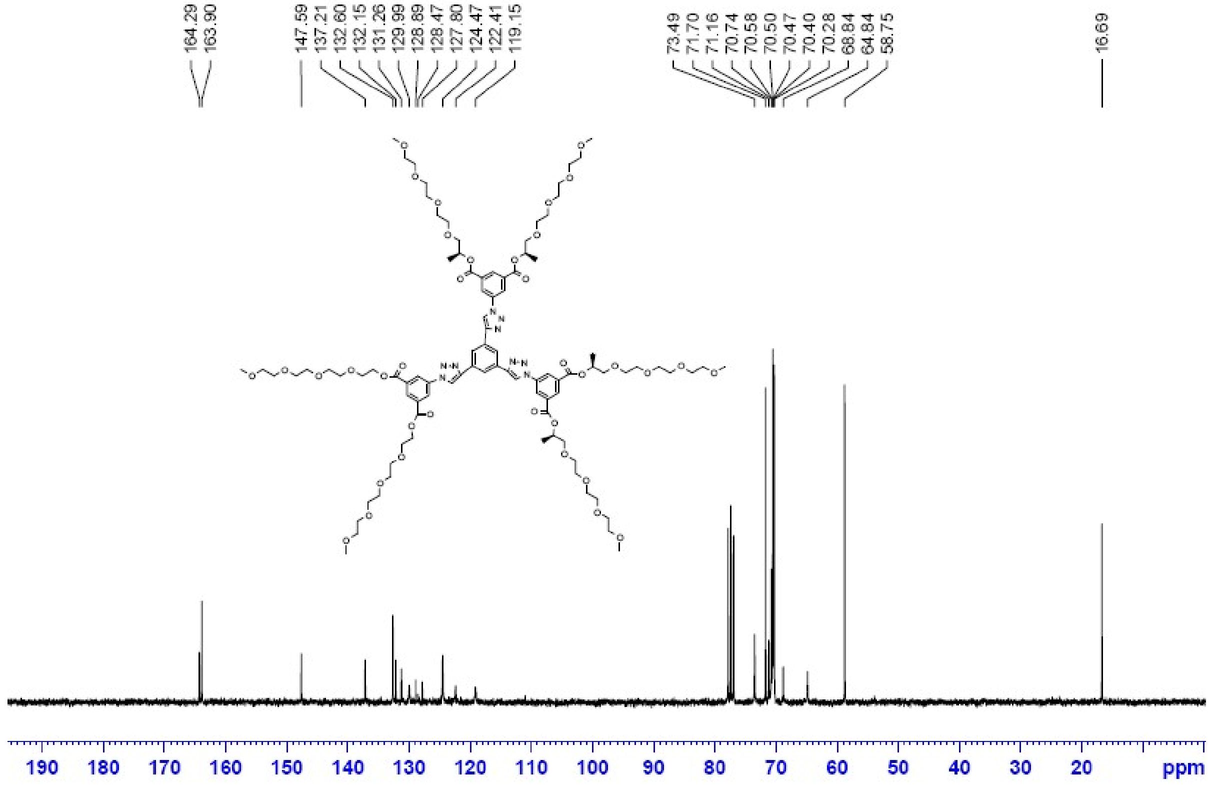


**Figure S10.** $^{13}C$ NMR spectrum of compound **8** (100 MHz, $CDCl_3$, 25 °C)

**Supplementary Table S1.** Per-residue binding free energy contributions (kcal/mol) of key amino acids to the binding of dendrimer 9 with the ERα receptor (AMBER system numbering).

| Residue (AMBER No.) | ΔG Contribution (kcal/mol) | Putative Corresponding Residue in PDB 3ERT[1] | Interaction Type[2] |
|---|---|---|---|
| Asp 6 | -6.50 ± 0.5 | Asp 311 | Electrostatic / H-bond |
| Val 63 | -5.87 ± 0.4 | Val368 | Hydrophobic |
| Leu 65 | -5.24 ± 0.4 | Leu 370 | Hydrophobic |
| Thr 66 | -6.89 ± 0.5 | Thr 371 | H-bond / Polar |
| Leu 67 | -10.14 ± 0.7 | Leu 372 | Hydrophobic |
| Asp 69 | -4.24 ± 0.3 | Asp 374 | Electrostatic |
| Gln 70 | -7.61 ± 0.5 | Gln 375 | H-bond / Polar |
| Val 71 | -5.49 ± 0.4 | Val 376 | Hydrophobic |
| Ser 163 | -6.53 ± 0.5 | Ser 450 | H-bond / Polar |
| Glu 165 | -5.27 ± 0.4 | Glu 452 | Electrostatic |
| Lys 167 | -8.76 ± 0.6 | Lys 454 | Electrostatic / Cation-π |
| Arg 243 | -6.47 ± 0.5 | Arg 548 | H-bond / Electrostatic |
| Leu 244 | -6.14 ± 0.5 | Leu 549 | Hydrophobic |
| Pro 247 | -7.24 ± 0.5 | Pro 552 | Hydrophobic |

**Footnotes for Supplementary Table S1:**

[1] **Putative Corresponding Residue in PDB 3ERT:** Due to the automated preprocessing and solvation box setup in AMBER tleap, the residue numbering in the simulation system differs from the canonical PDB (3ERT) numbering. A full residue mapping table between the AMBER system and PDB 3ERT is available from the authors upon request.

[2] **Interaction Type:** Suggested primary non-covalent interaction based on residue chemistry and decomposed energy terms (van der Waals vs. electrostatic). Specific interactions are detailed in the main text (Figure 5, ligand-residue interaction profiles).
